# Natural Trienoic Acids as Anticancer Agents: First Stereoselective Synthesis, Cell Cycle Analysis, Induction of Apoptosis, Cell Signaling and Mitochondrial Targeting Studies

**DOI:** 10.3390/cancers13081808

**Published:** 2021-04-10

**Authors:** Vladimir A. D’yakonov, Alexey A. Makarov, Lilya U. Dzhemileva, Ilfir R. Ramazanov, Elina Kh. Makarova, Usein M. Dzhemilev

**Affiliations:** Institute of Petrochemistry and Catalysis, Russian Academy of Sciences pr. Oktyabrya 141, 450075 Ufa, Russia; makarovalexink@yandex.ru (A.A.M.); ilfir.ramazanov@gmail.com (I.R.R.); makarovaelina87@yandex.ru (E.K.M.); dzhemilev@anrb.ru (U.M.D.)

**Keywords:** Z,Z,Z-trienoic acids, cross-cyclomagnesiation, topoisomerase inhibitors, anticancer activity, apoptosis, cell signaling

## Abstract

**Simple Summary:**

Currently, the whole world is acutely concerned with the selection of effective treatment regimens for oncological diseases. This problem is becoming more and more catastrophic every year due to the phenomenon of multidrug resistance, the consequence of which is the loss of the effectiveness of drugs against tumor cells. One of the solutions to the problem described above is the synthesis of new low molecular weight compounds that can effectively affect molecular cellular targets, for example, enzymes of the cell cycle, and, as a consequence, interrupt DNA synthesis, contributing to tumor death. Within the framework of this article, we carried out the Z-stereoselective synthesis of natural unsaturated acids containing a 1Z,5Z,9Z-triene moiety, for which it was shown that they are effective inhibitors of human topoisomerase I, and also affect mitochondria. At the same time, using multiplex analysis, the activation of signaling pathways was studied and a probable mechanism of the antitumor action of the synthesized trienoic acids was proposed.

**Abstract:**

The first Z-stereoselective method was developed for the synthesis of unsaturated acids containing a 1Z,5Z,9Z-triene moiety in 61–64% yields using the new Ti-catalyzed cross-coupling of oxygen-containing and aliphatic 1,2-dienes as the key synthetic step. It was shown for the first time that trienoic acids with non-methylene-interrupted Z-double bonds show moderate cytotoxic activities against tumor cell lines (Jurkat, K562, U937, HL60, HeLa), human embryonic kidney cells (Hek293), normal fibroblasts and human topoisomerase I (hTop1) inhibitory activity in vitro. The synthesized acids efficiently initiate apoptosis of Jurkat tumor cells, with the cell death mechanism being activated by the mitochondrial pathway. A probable mechanism of topoisomerase I inhibition was also hypothesized on the basis of in silico studies resorting to docking. The activation and inhibition of the most versatile intracellular signaling pathways (CREB, JNK, NFkB, p38, ERK1/2, Akt, p70S6K, STAT3 and STAT5 tyrosine kinases) responsible for cell proliferation and for initiation of apoptosis were studied by multiplex assay technology (Luminex xMAP).

## 1. Introduction

According to reports and predictions of the World Health Organization, cancer diseases permanently rank first among human fatal diseases and this sad statistic does not tend to improve in the near future (Causes of Death. Available online: https://ourworldindata.org/causes-of-death, accessed on 8 April 2021). The mortality rate caused by malignant neoplasms is also very high; now, the worldwide average is 150 per 100,000 population. By as soon as 2030, the number of people diagnosed with cancer may increase 1.5-fold. About 10,000 scientific publications dealing with cancer diagnosis, treatment and prevention appear every month, which confirms high importance and relevance of this problem.

In this regard, a necessary condition for the search for new highly effective low-toxic drugs is the development of compounds with a clearly defined effect on the molecular targets of carcinogenesis. In the context of the above, human topoisomerases I (hTopI) and II (hTop2α), which are one of the key enzymes of DNA synthesis, are promising molecular targets for new generation anticancer drugs.

Currently, quite a few compounds of various classes able to inhibit topoisomerases are known. Some of these compounds are isolated from natural objects, e.g., camptothecin, podophyllotoxin, anthracyclines, polyenoic acids and many other.

Among numerous classes of compounds, polyunsaturated fatty acids attract special attention of researchers, as they are significant for the vital activity of all living organisms, as they are precursors of potent eicosanoid bioregulators such as prostaglandins and leukotrienes [1,2,3,4], are involved in lipid metabolism [5], exhibit antihistaminic [6] and anti-inflammatory effects [7], possess antitumor properties [8,9,10] and stimulate immune response [11]. They are widely used for prevention and treatment of cardiovascular diseases [11,12,13] and are promising for the treatment of brain diseases [14].

**Figure 1:**
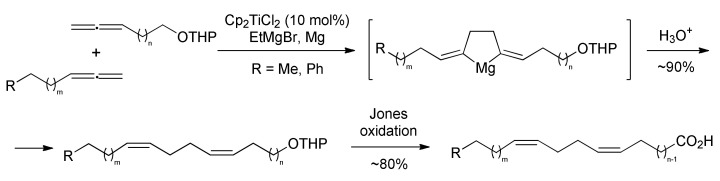
Stereoselective synthesis of dienoic acids.

Among the whole diversity of polyunsaturated acids, of most interest, in our opinion, are non-methylene-interrupted fatty acids (up to C34), which have unique structure and are found in mollusks [15,16], sea sponges [17,18] and gymnosperm seeds [19,20]. The most recent studies demonstrated the efficiency of these compounds as antimicrobial [21], antibacterial, antimalarial, antifungal, anti-inflammatory and trypanocidal and anti-leishmaniasis agents [7,22]. In addition, several research groups performed intense studies of the biomedical potential of 5*Z*,9*Z*-dienoic acids acting as topoisomerase inhibitors. Topoisomerases are a class of enzymes called isomerases that have a marked effect on DNA topology. Topoisomerases are able to relax supercoiled DNA molecules, thereby affecting transcription, replication and recombination and are considered by modern pharmacology as molecular targets for antitumor agents [23,24,25,26,27]. At the same time, the high complexity of the isolation of natural 5*Z*,9*Z*-dienoic acids, as well as their extremely low concentration in natural objects, greatly complicate and often make it almost impossible to establish a relationship between their biological activity and structure. Earlier, in our laboratory, we developed an original stereoselective method for the synthesis of fatty acids with a given position of the 1*Z*,5*Z*-diene group relative to the carboxyl group. The key step in this synthetic pathway of aliphatic and oxygen-containing 1,2-dienes was a new catalytic reaction of intermolecular cross-cyclomagnesiation [28] (Figure 1).

**Figure 2:**
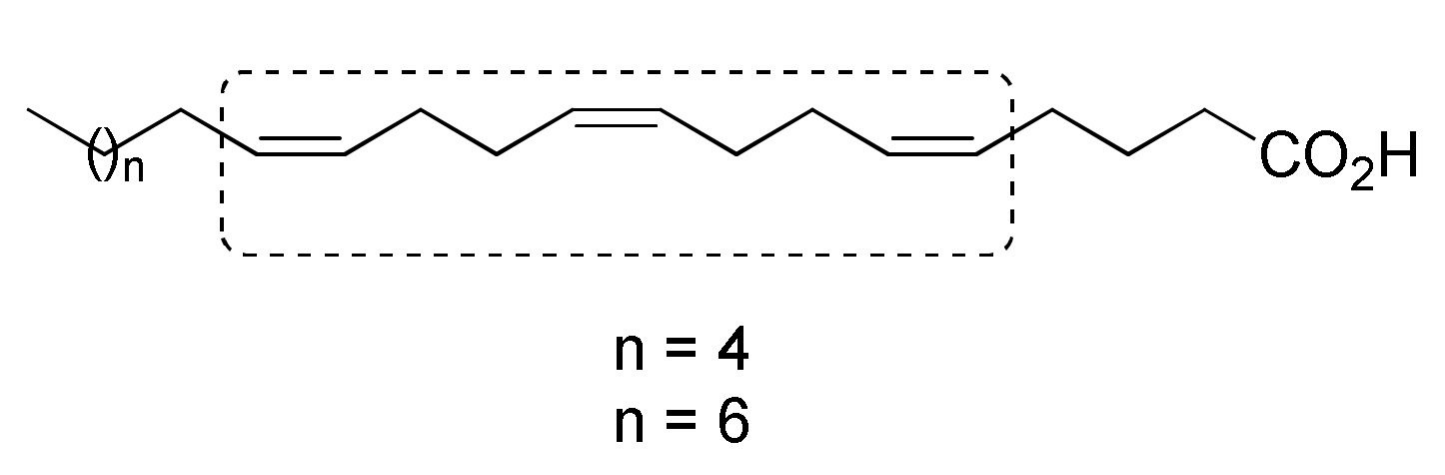
Natural 1Z,5Z,9Z-trienoic acids from the sea anemone *S. helianthus*.

In continuation of these studies, we assumed that this method of synthesis of unsaturated acids could be extended to unique trienoic acids containing a 1*Z*,5*Z*,9*Z*-triene moiety in the molecule, particularly, natural acids that were found in phospholipids of the sea anemone *Stoichactis helianthus* [29] (Figure 2).

The minor content of these acids in natural products and the difficulty of isolation are the key factors that hamper investigations of their biomedical potential.

Therefore, development of stereoselective methods for the preparative synthesis of natural trienoic acids and studies of the biological activities of the products were among the primary goals.

## 2. Results

### 2.1. Chemistry

**Scheme 1:**
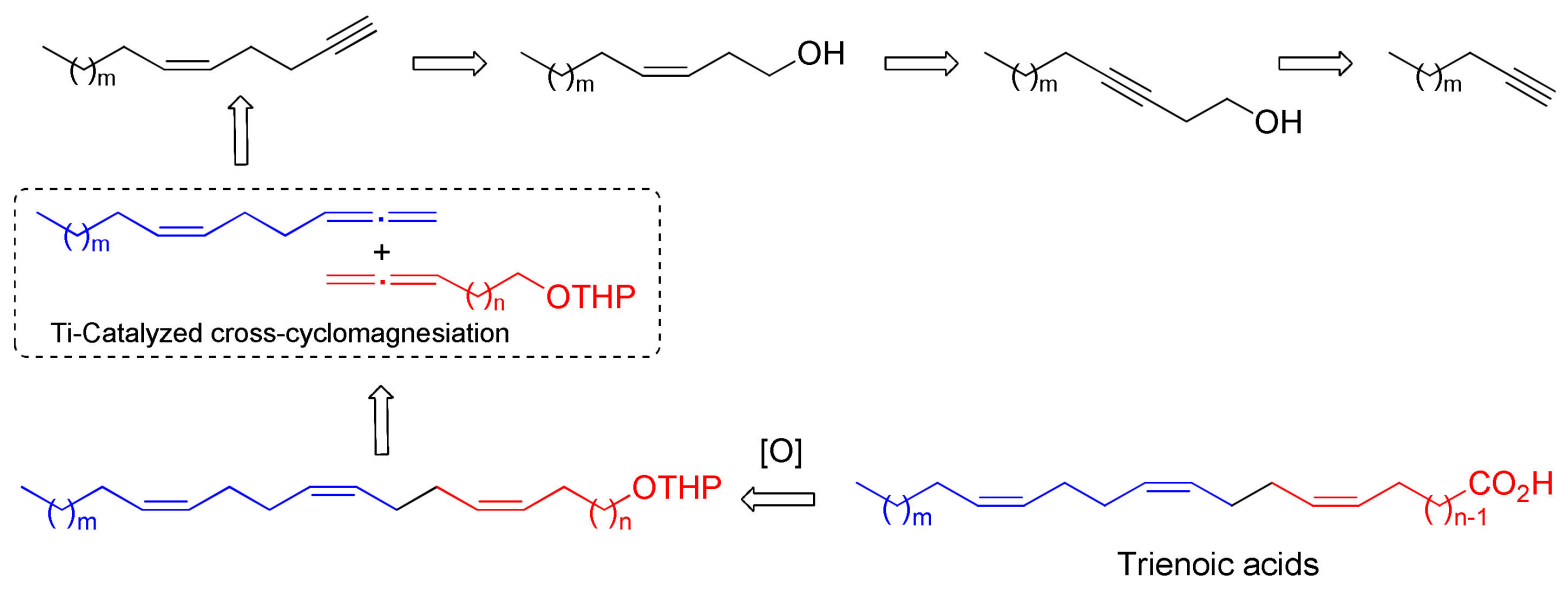
Retrosynthetic analysis of the synthesis of natural trienoic acids.

Our study started with the retrosynthetic analysis of the structure of trienoic acids with non-methylene-interrupted *Z*-double bonds, which demonstrated that the total synthesis can be implemented through Ti-catalyzed cross-cyclomagnesiation of (6*Z*)-alka-1,2,6-trienes with tetrahydropyran ethers of allene alcohols (Scheme 1). The final step of the synthesis of the desired acids is the Jones oxidation of the trienol tetrahydropyran ether that is formed upon acid hydrolysis of the reaction mixture (Scheme 1).

**Scheme 2:**
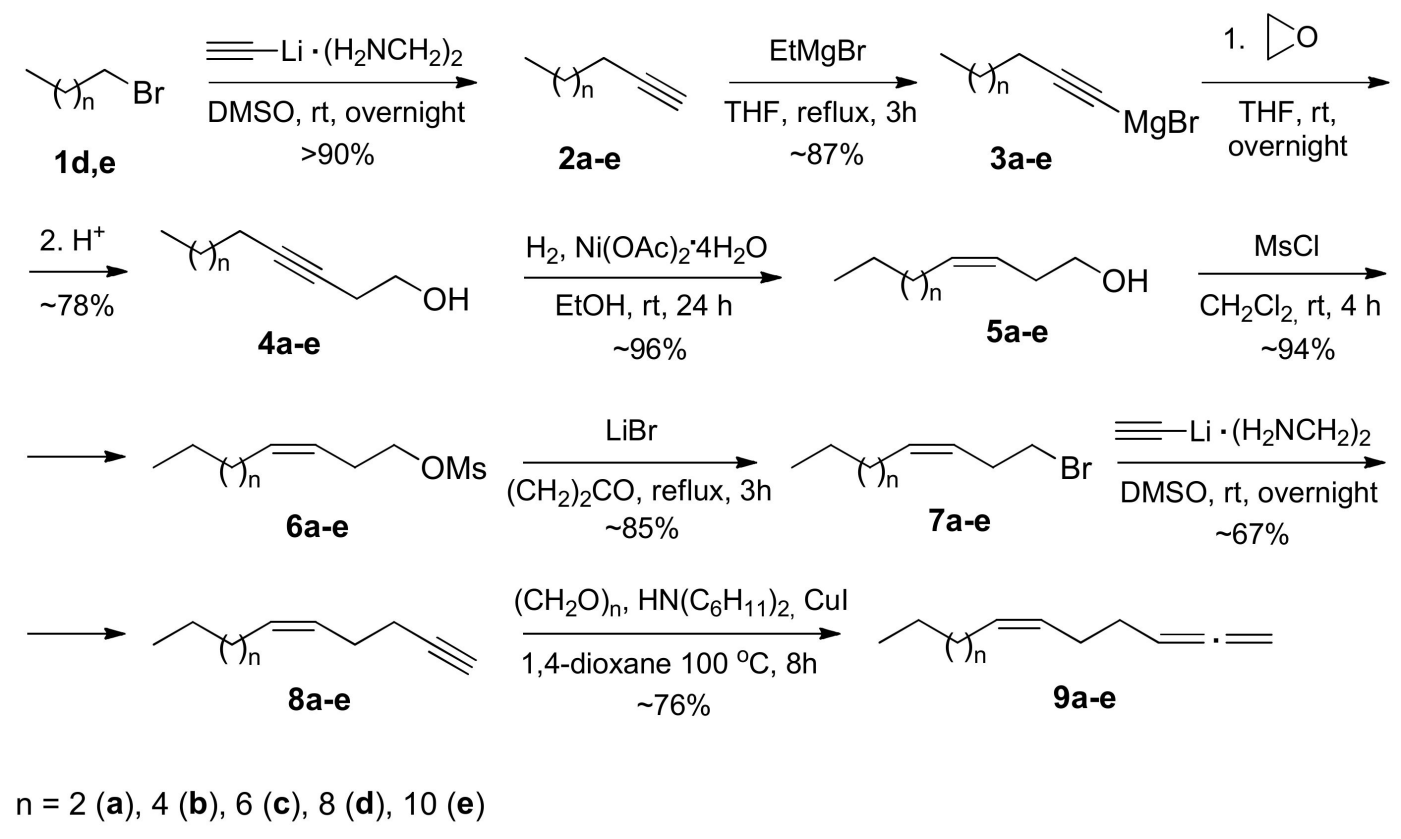
Synthesis of (6*Z*)-alka-1,2,6-trienes (**9a**–**e**).

It is noteworthy that the synthesis of key monomers, (6Z)-alka-1,2,6-trienes, was a separate task. Therefore, we developed an approach to these compounds based on commercially available 1-bromoalkanes (1-bromodecane, 1-bromododecane) and 1-alkynes (1-hexyne, 1-octyne, 1-decyne) (Scheme 2). This eight-step method includes the reaction of 1-bromoalkanes **1d**,**e** with the lithium acetylide ethylenediamine complex in dimethyl sulfoxide (DMSO), which gives 1-alkynes **2d**,**e** in >90% yields. The subsequent treatment of the resulting compounds **2d**,**e** or commercially available 1-alkynes **2a**–**c** with EtMgBr in THF and refluxing for 3 h affords magnesium acetylides **3a**–**e**, which are then allowed to react with ethylene oxide. The subsequent acid hydrolysis leads to alk-3-yn-1-ols **4a**–**e**. Then, (3*Z*)-alk-3-en-1-ols **5a**–**e** are prepared by Ni(OAc)-catalyzed hydrogenation of alkynols 4a–e; the resulting alcohols **5a**–**e** are treated with methanesulfonyl chloride in CH_2_Cl_2_ to afford mesylates 6a–e within 4 h. Refluxing of the latter with lithium bromide in acetone for 3 h results in the formation of 1-bromo-(3*Z*)-alk-3-enes **7a**–**e** in ~85% yields. At the final stage, alkynes **8a**–**e** are synthesized by the reaction of alkenyl bromides **7a**–**e** with lithium acetylide ethylenediamine complex, while the acetylene-allene rearrangement of alkynes **8a**–**e** under the Mannich reaction conditions yields the target (6*Z*)-alka-1,2,6-trienes **9a**–**e** (Scheme 2).

**Scheme 3:**
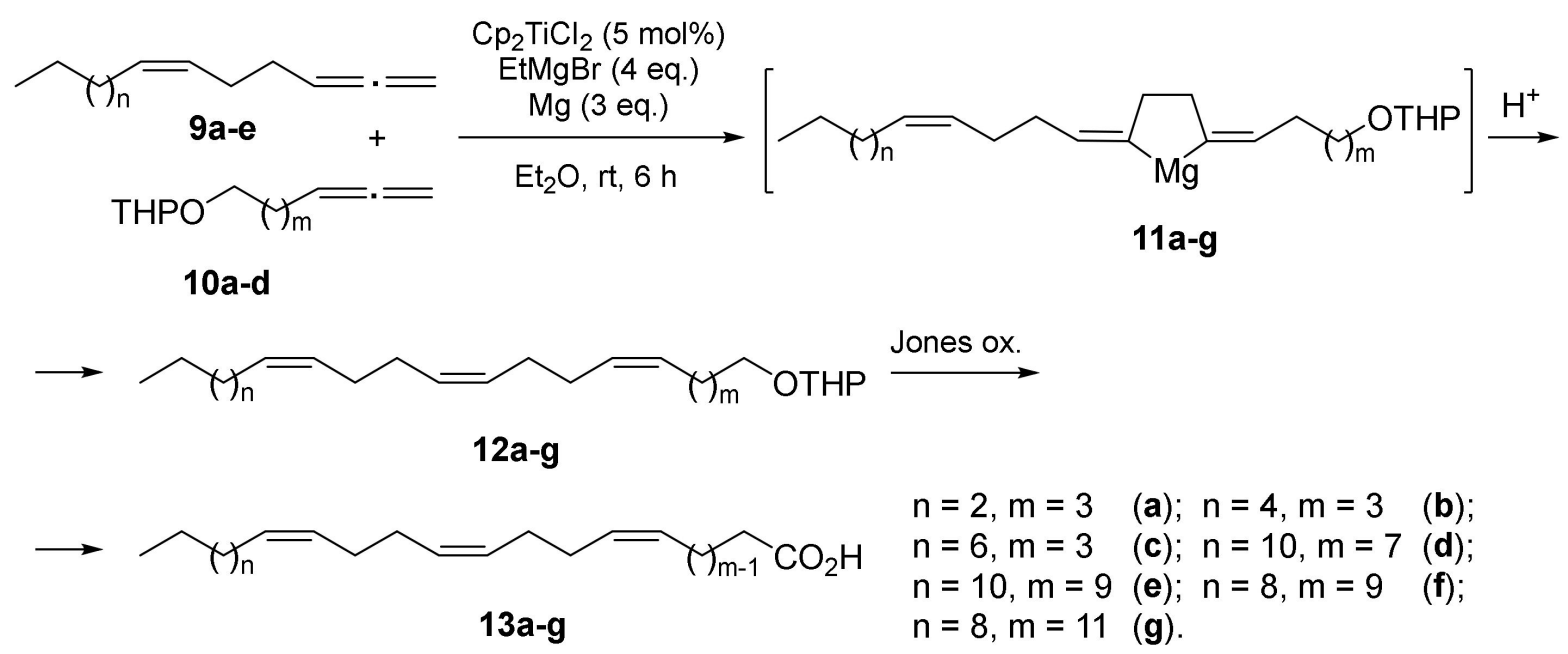
(*Z*,*Z*,*Z*)-Stereoselective synthesis of trienoic acids **13a**–**g**.

According to the synthetic route to 1*Z*,5*Z*,9*Z*-trienoic acids (5*Z*,9*Z*,13*Z*-octadecatrienoic **13a**, 5*Z*,9*Z*,13*Z*-eicosatrienoic **13b**, 5*Z*,9*Z*,13*Z*-docosatrienoic **13c**, 9*Z*,13*Z*,17*Z*-triacontatrienoic **13d**, 11*Z*,15*Z*,19*Z*-dotriacontatrienoic **13e**, 11Z,15Z,19Z-triacontatrienoic **13f** and 13*Z*,17*Z*,21*Z*-dotriacontatrienoic **13g** acids) (Scheme 3), developed on the basis of retrosynthetic analysis, we carried out intermolecular cross-cyclomagnesiation of (6*Z*)-alka-1,2,6-trienes **9a**–**e** ((6*Z*)-undeca-1,2,6-triene **9a**, (6*Z*)-trideca-1,2,6-triene **9b**, (6*Z*)-pentadeca-1,2,6-triene **9c**, (6*Z*)-heptadeca-1,2,6-triene **9d**, (6*Z*)-nonadeca-1,2,6-triene **9e**) with allene alcohol tetrahydropyran ethers **10a-d** on treatment with EtMgBr in the presence of Mg (powder) and catalytic amounts of Cp_2_TiCl_2_ (**9a**–**e**:**10a**–**d**:EtMgBr:Mg:[Ti] = 12:10:36:24:0.1, Et_2_O, 20–22 °C, 10 h). The in situ acid hydrolysis of magnesacyclopentanes **11a**–**g** thus formed gave tetrahydropyran ethers **12a**–**g** with 1*Z*,5*Z*,9*Z*-triene moieties in 81–89% yields (Scheme 3). Finally, the Jones oxidation of ethers **12a**–**g** furnished the target acids **13a**–**g** in 61–64% yields (Scheme 3).

**Scheme 4:**
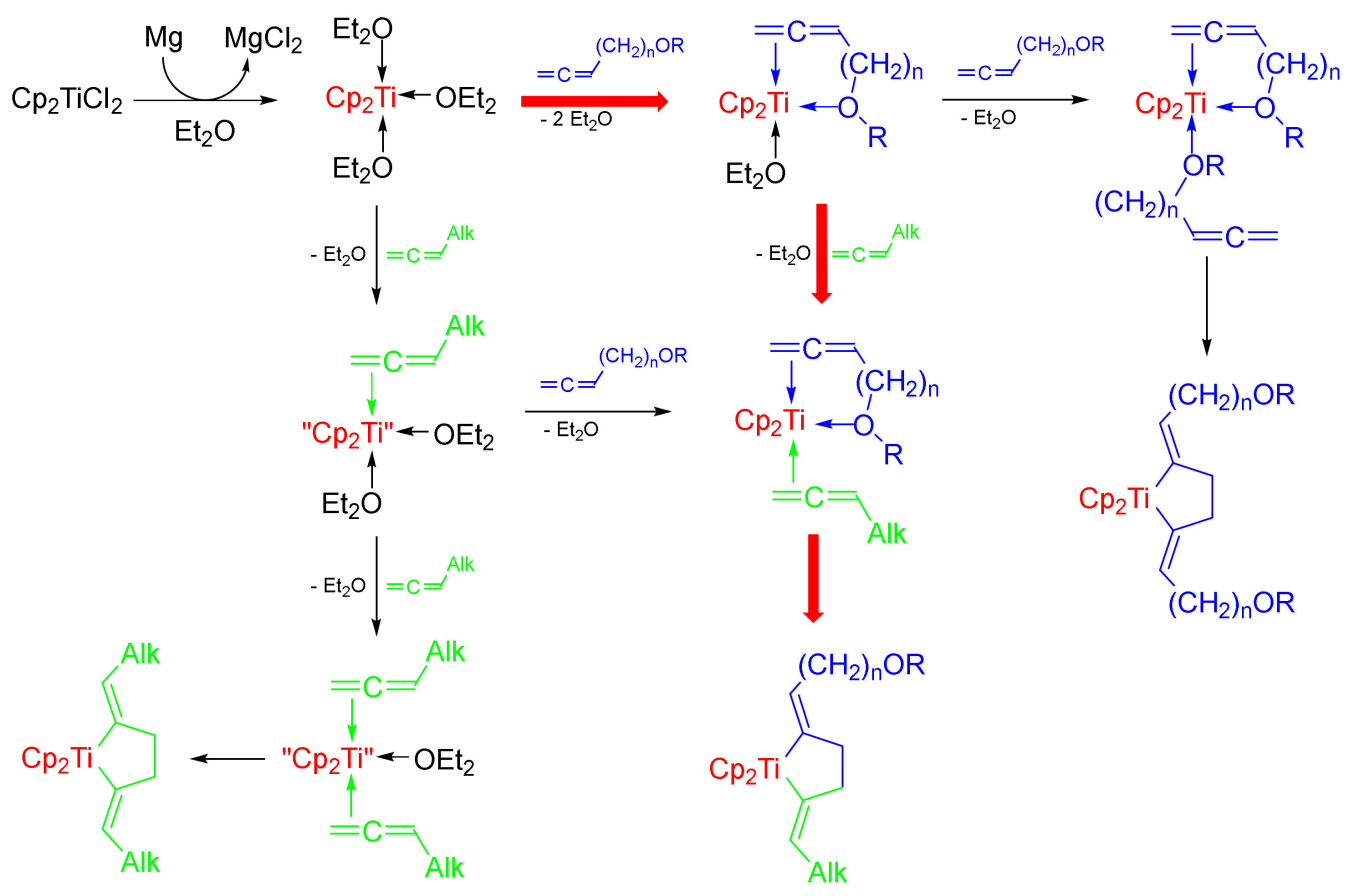
Putative scheme of the predominant formation of cross-cyclomagnesiation products from aliphatic and oxygen-containing allenes.

An unusual fact is that a mixture of alkyl-substituted allene with the tetrahydropyran ether of allene alcohol selectively gives the cross-coupling product under the reaction conditions. Meanwhile, these allene derivatives taken in a pure state without the partner are smoothly converted to homo-coupling products. This intriguing feature of this system, which allowed us to successfully prepare dienoic and trienoic acids, can be explained in the following way:

Titanocene generated upon the reduction of titanocene dichloride is a coordinatively unsaturated low-valence titanium complex. In our opinion, because of the coordinative unsaturation, titanocene forms a stronger complex with the bidentately coordinated tetrahydropyran ether of allene alcohol than with alkyl-substituted allene. The complex thus formed is already largely coordinatively saturated because of the strong binding of titanium to the allene double bond. Therefore, this complex would react at a higher rate with more nucleophilic alkyl-substituted allene. Meanwhile, the presence of oxygen in the tetrahydropyran ether of allene alcohol decreases the double bond nucleophilicity and, hence, reactivity towards the addition of the second allene molecule to titanocene. Thus, the formation of the cross-coupling product is quite expectable, if one takes into account the nature and the energy of the frontier orbitals of the intermediate titanocene (Scheme 4).

Thus, the key point is the original (*Z*,*Z*,*Z*)-stereoselective total synthesis of trienoic acids, which contain the 1*Z*,5*Z*,9*Z*-triene system, developed by us, using a new Ti-catalyzed cross-cyclomagnetation 1,2-dienes with readily available Grignard reagents. This method is an important step for the development of new highly effective anticancer drugs.

### 2.2. Biological Evaluation

#### 2.2.1. Cytotioxic Activity In Vitro

Trienoic acids **13a**–**g**, which we synthesized, were evaluated for antitumor activity in vitro using the Jurkat, K562, U937, HL60, HeLa and HEK293 cell lines and normal fibroblasts (Table 1). The assays included determination of IC_50_ by flow cytofluorimetry.

The assays showed that trienoic acids **13a**–**g** exhibit a cytotoxic effect against the chosen cell lines, which is comparable with or somewhat higher than the effect of the previously synthesized 5*Z*,9*Z*-dienoic acids. Acid **13c** had the highest cytotoxicity against all of the cell lines chosen for the assays. The selectivity index (SI) of the therapeutic and cytotoxic action for the Jurkat, K562, U937 and HL60 cell lines was 4–9 times higher than that for normal fibroblasts and conditionally normal human embryonic kidney cells Hek293 (Table 1).

#### 2.2.2. Apoptosis and Cell Cycle Research

**Figure 3:**
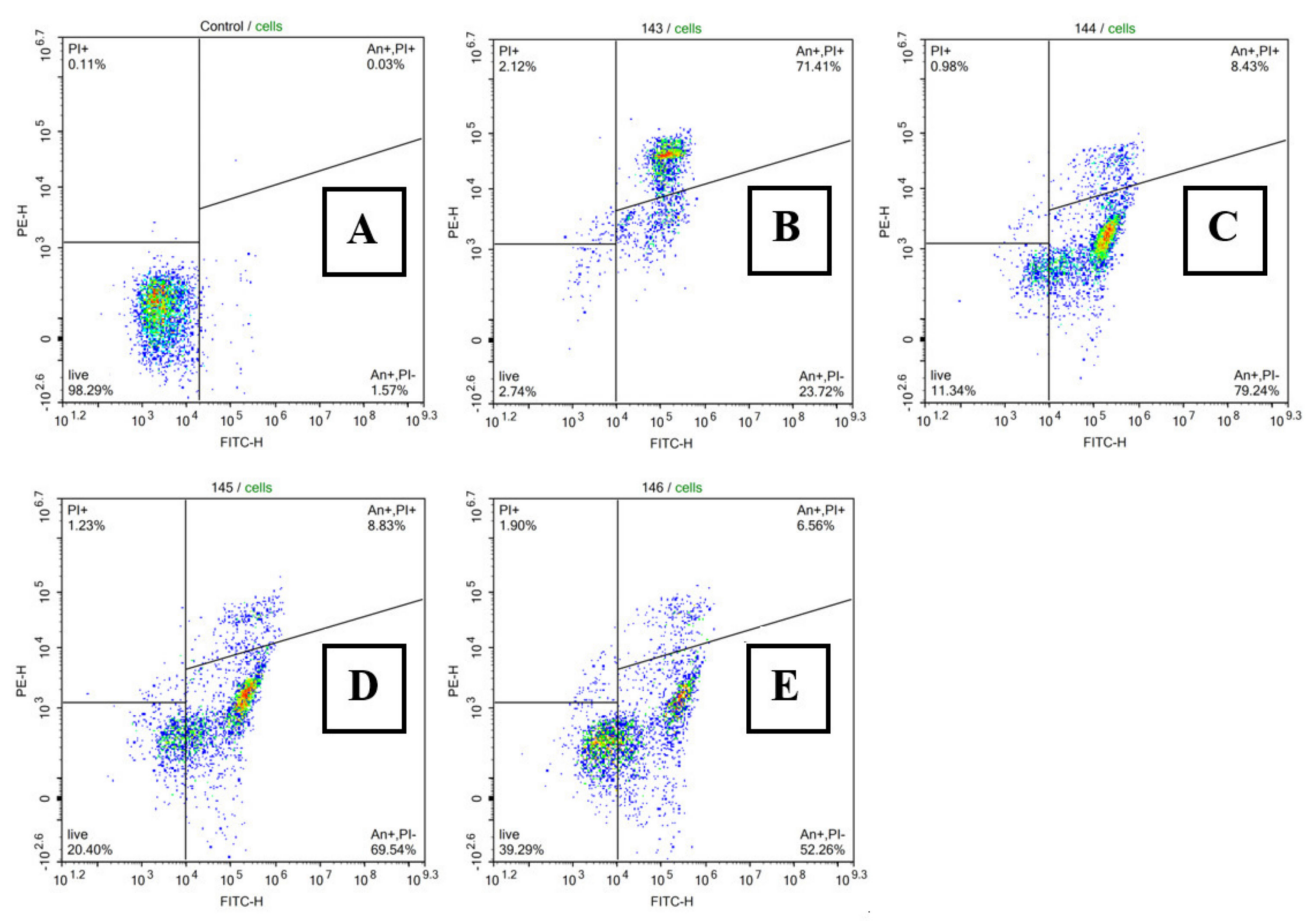

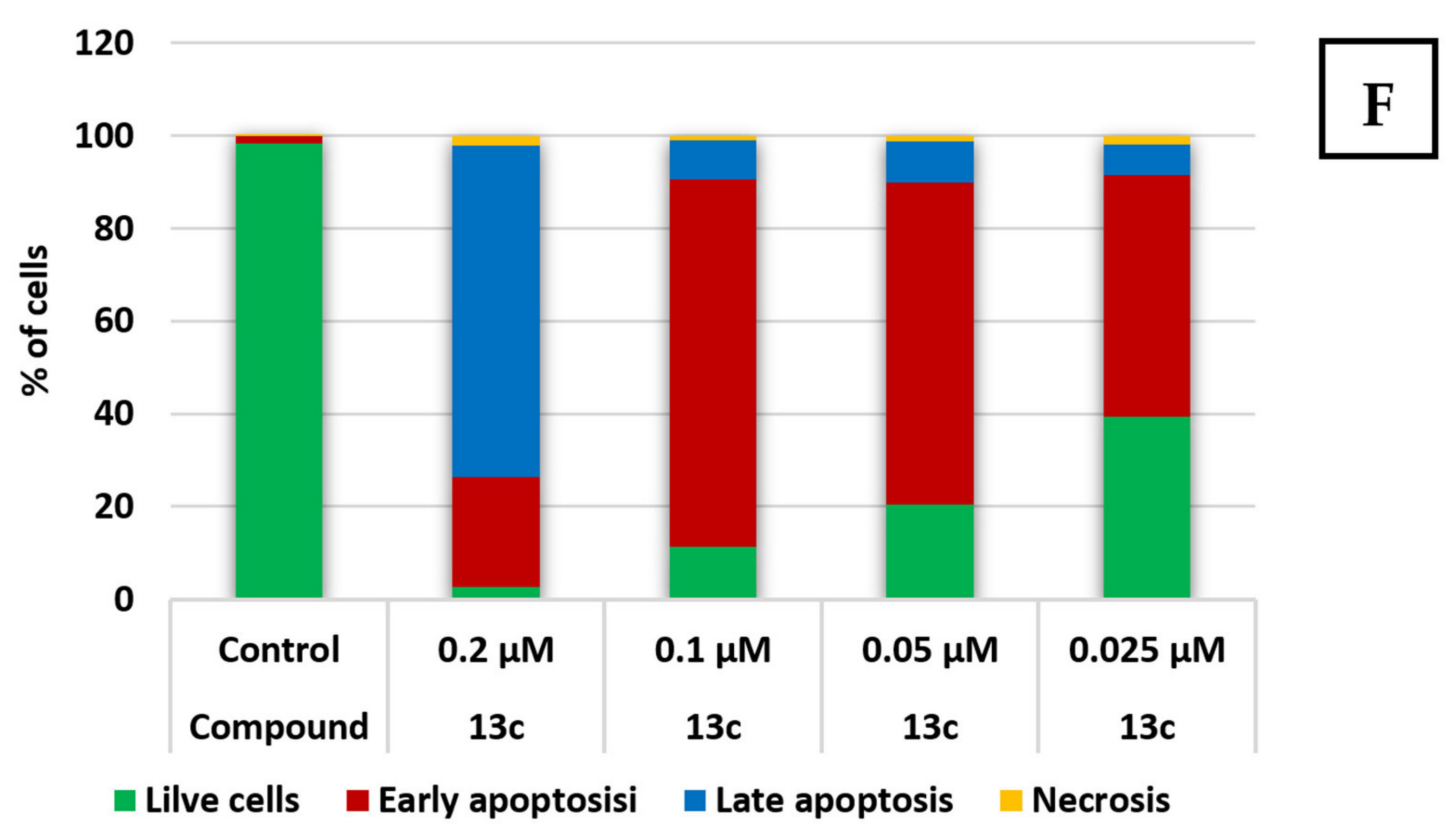
Analysis of the induction of apoptosis in Jurkat cells after exposure to compound **13c** in various concentrations. (**A**) control sample untreated with the test compound; (**B**) compound **13c** in 0.2 µM concentration; (**C**) compound 1**3c** in 0.1 µM concentration; (**D**) compound **13c** in 0.05 µM concentration; (**E**) compound **13c** in 0.025 µM concentration; (**F**) histogram of apoptosis phases in Jurkat cells treated with compound **13c**. The cells treated with compound 13c in various concentrations were stained with Annexin V/7-AAD. The incubation time of compound **13c** with the cells was 24 h.

In accordance with the results of the cytotoxicity of the studied compounds, we more deeply assessed the ability of compound **13c** to induce apoptosis in Jurkat cell culture and to influence the phases of the cell cycle (Figure 3).

The effect of compound **13c** resulted in apoptosis of Jurkat cells and this effect was comparable to that of camptothecin and etoposide, the popular cytostatic agents in medicine. The highest rate of apoptosis in Jurkat cells (71.41%) was the exposure to test compound **13c** at a concentration of 0.2 µM and a concentration of 0.025 µM caused the lowest percentage of Jurkat cells in the late apoptosis stage (6.56%) (Figure 3). High activity of compound **13c** towards live cells may be attributable to the presence of *cis*-double bonds in the molecule because, as already known, these compounds show high inhibitory activity against topoisomerase I.

**Figure 4:**
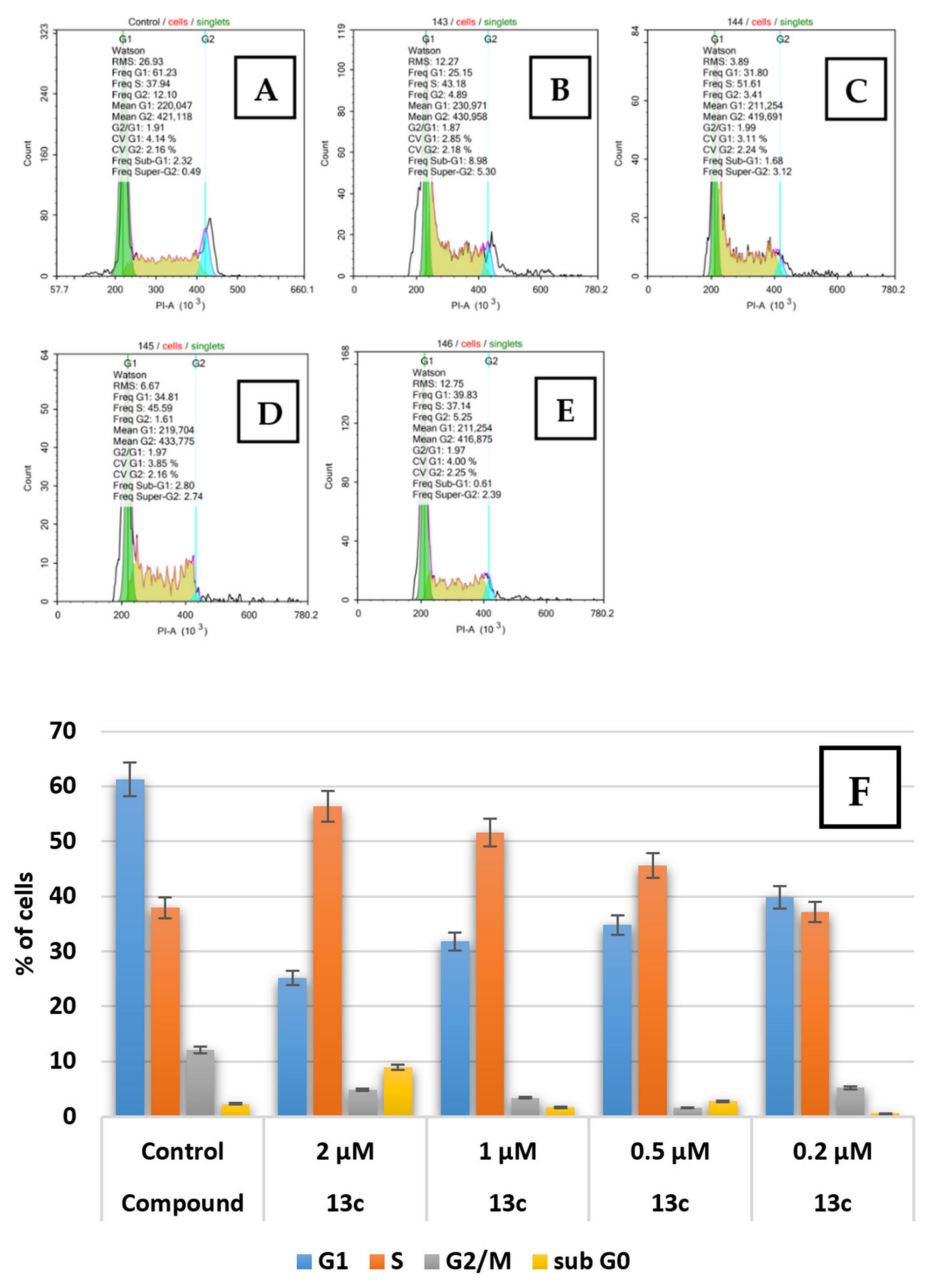
Cytofluorometric analysis of cell cycle phases (**A**–**E**) in Jurkat tumor cells treated with compound **13c**. The cells treated with various concentrations of **13c** and stained with PI. The incubation time of compound **13c** with the cells was 24 h. (**A**) Control sample untreated with the test compound; (**B**) Compound **13c** in 0.2 µM concentration; (**C**) Compound **13c** in 0.1µM concentration; (**D**) Compound **13c** in 0.05 µM concentration; (**E**) Compound **13c** in 0.025 µM concentration; (**F**) Histogram of cell cycle phases in Jurkat cells treated with compound **13c**. Results are expressed as mean ± SE (*n* = 3).

Jurkat cells in the control sample show the balance of all phases of the cell cycle with a predominance of cells in the G0–G1 phase (Figure 4). The analysis of the cell cycle with the addition of compound **13c** to the Jurkat cell culture demonstrates a significant predominance of the population of hypodiploid cells (sub-G0-G1), a dramatic increase in the S-phase and a pronounced decrease in the percentage of G2 + M cells, which corresponds to the standard picture of the effect of classical cytostatics-camptothecin and etoposide. In summary, it can be argued that the compound **13c** has antitumor activity against cell T cell leukemia caused by apoptosis-inducing activity.

#### 2.2.3. Topoisomerase I Inhibition Assay and Molecular Docking Studies

**Figure 5:**
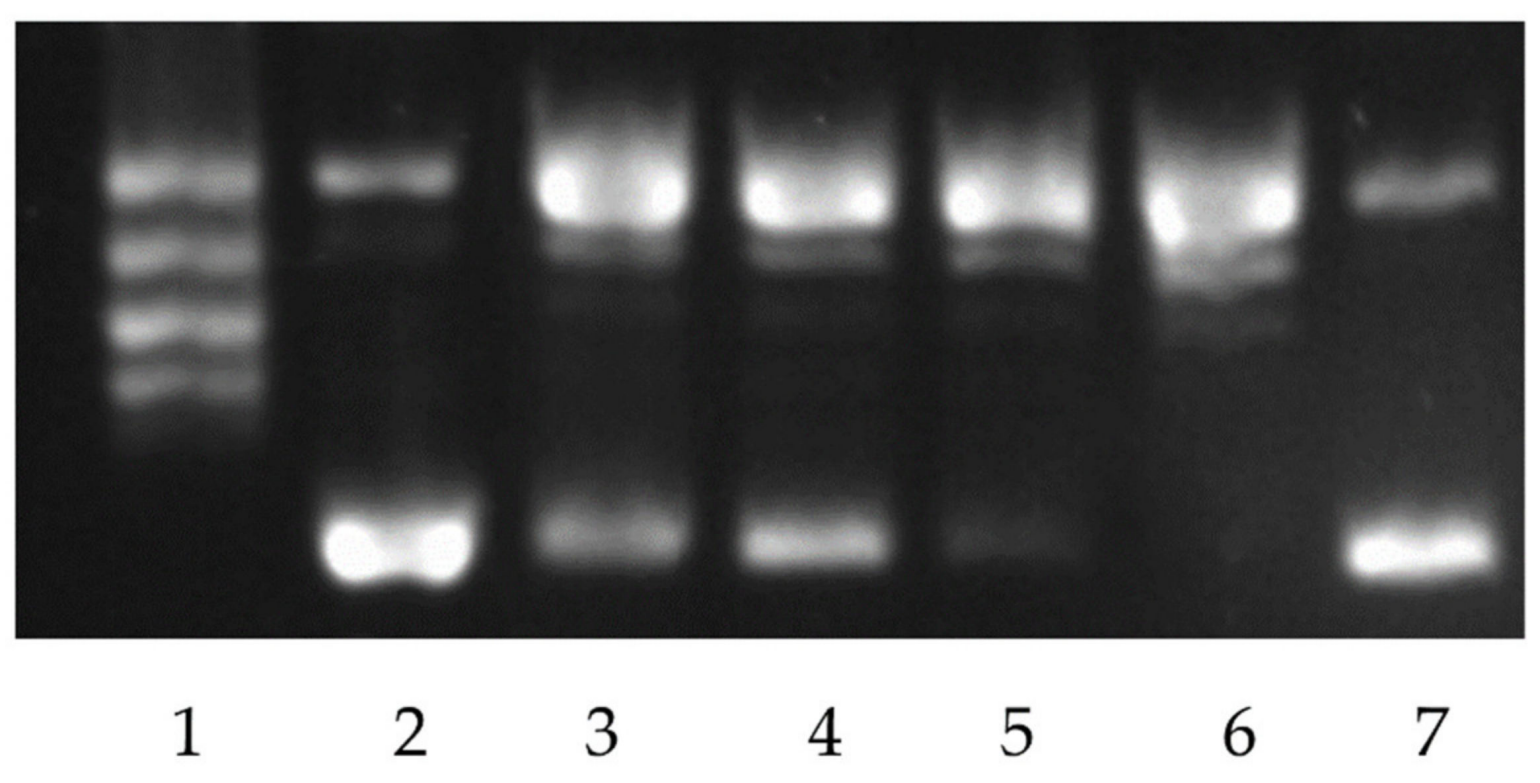
Electrophoretic separation in 2% agarose gel of the relaxation products of 250 ng of plasmid DNA (pHOT1) in vitro under the action of 4 U of topoisomerase I (TG-1018-2 Topoisomerase I Drug Screening Kit, Topogen, Buena Vista, CO, USA) in the presence of natural 5*Z*,9*Z*,13*Z*-docosatrienoic acid **13c** (the test compounds were added before the addition of topoisomerase I). (Lane 1) Relax DNA; (Lane 2) Supercoiled DNA; (Lane 3) **13c**, 0.1 μM; (Lane 4) **13c**, 0.05 μM; (Lane 5) **13c**, 0.02 μM; (Lane 6) No compound; (Lane 7) Camptotecin, 0.5 μM.

The ability of trienoic acids **13a**–**g** (Table 2) to interact with topoisomerase I in vitro with the formation of relaxation of the supercoiled plasmid pHOT1 was studied (Figure 5).

The data of this study, showing the dependence of the amount of relaxed DNA on the structure of the obtained trienoic acids, will serve as further chemical modifications that will significantly improve the chemotherapeutic properties of these fatty acids (Figure 5).

The addition of trienoic acids to the relaxation reaction of supercoiled DNA at a concentration of IC_50_ makes it possible to detect a gradual decrease in the formed topoisomers and, as a consequence, to observe an increase in the open circular form of the plasmid, which directly indicates a decrease in the enzyme activity. When no test compound was added, no such effect was observed (Figure 5, line 6). All seven unsaturated acids we prepared behaved almost identically in the given concentration range; they started to inhibit topoisomerase I when present in concentrations below 0.1–0.8 µM. A high inhibitory activity against human topoisomerase I was found for 5*Z*,9*Z*,13*Z*-docosatrienoic acid **13c** in concentrations above 0.1 μM. Thus, we found that even micromolar concentrations of compounds **13a**–**g** are able to suppress the catalytic activity of topoisomerase I.

**Figure 6:**
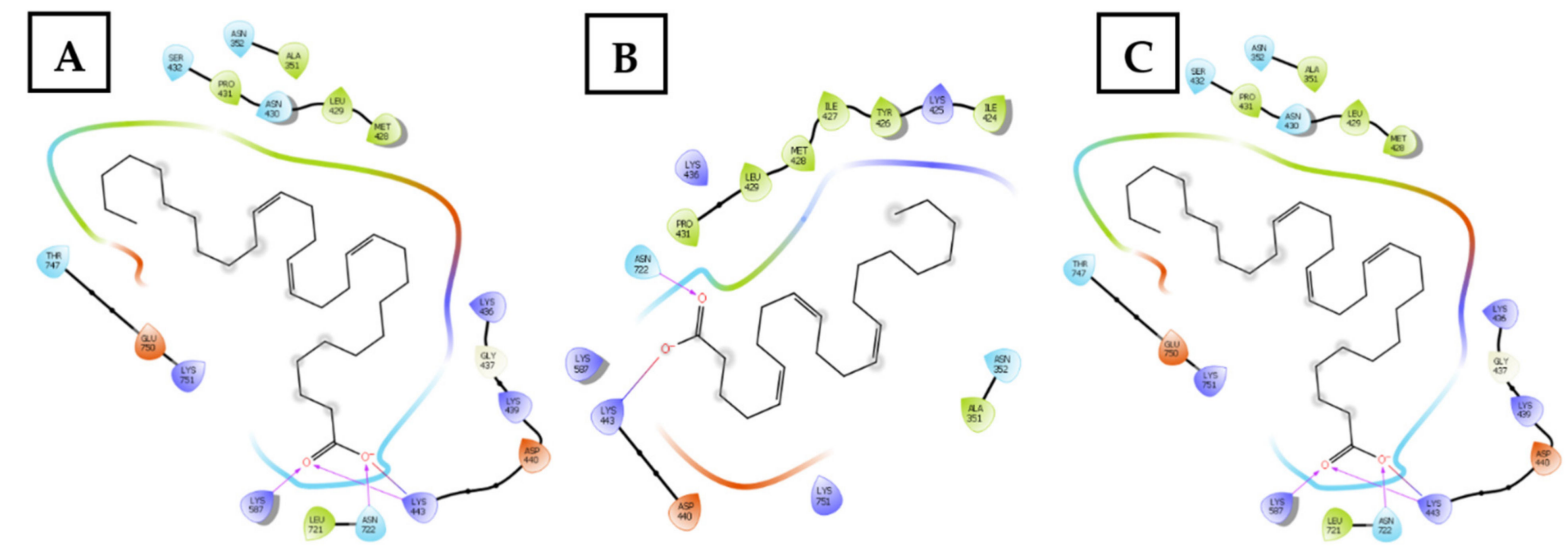
Ligand interaction diagram for acids **13b** (**A**), **13c** (**B**) and **13f** (**C**).

Molecular dynamics studies of the mechanism of topoisomerase I inhibition by eicosapentaenoic acid (cEPA) showed that the K443, K587 and N722 residues are the preferable binding sites (Figure 6) [30]. Presumably, the interaction of the carboxylate group of unsaturated carboxylic acids with the above-mentioned sites of Topo I gives a stable complex, which prevents the catalytic center at Y723 from performing a nucleophilic attack on the DNA phosphate. Similar results were obtained by molecular docking for the mechanism of topoisomerase I inhibition by unsaturated cholesterol-containing acids and synthetic analogues of natural 5Z,9Z-dienoic acids [27,31]. It can be assumed that synthetic Z,Z,Z-trienoic acids **13a**–**g** should inhibit human Topo I by similar mechanisms. A truncated hTopI form (70 kDa) complexed with the 22-pair duplex oligonucleotide (PDBID 1A36), from which the duplex oligonucleotide fragment and water molecules were removed, served as the initial model for the molecular docking (Figure 6). The interaction of synthetic Z,Z,Z-trienoic acids **13a**–**g** with the 1A36 fragment were analyzed using the OPLS3 method (Glide XP). We found that the preferable binding sites for the carboxylate group in the compounds **13a**–**g** are the K443, K587 and N722 residues. The docking score calculated by the OPLS3 (Glide XP) method increases in the following series of compounds: **13b** (−7.5) < **13c** (−6.8) < **13e** (−5.9) < **13g** (−5.1) < **13a** (−4.6) < **13d** (−4.4) < **13f** (−2.9), which is generally correlated with the experimentally observed activities of the synthetic Z,Z,Z-trienoic acids.

#### 2.2.4. Studying the Effect of Trienoic Acids on Mitochondria

Mitochondria are extremely important as the main organelles that ensure the energy balance of the cell. Moreover, they regulate a number of apoptotic and proapoptotic proteins, are the main source of ATP and interact with reactive oxygen species (ROS). Today mitochondria are key organelles involved in the pathogenesis of many forms of cancer, inflammatory and neurodegenerative processes [32].

The mitochondrial regulation of apoptosis and permeability of mitochondrial membrane upon variation of the potential difference between the inner and outer membrane parts are closely interrelated, which has been demonstrated by a large number of research groups all over the world. According to Mitchell’s theory, the proton electrochemical potential ΔΨ, arising during the transfer of electrons through the inner membrane, determines the interaction of oxidation and phosphorylation processes, the conjugation of which serves as a source of energy for the formation of adenosine triphosphate [33]. Various changes in ΔΨ detected by the MitoSense Red dye, in combination with the externalization of phosphatidylserine on the cell membrane surface, is a reliable sign of apoptosis due to mitochondrial damage and the formation of ROS ions. The expression of phosphotidylserine on the membrane is assessed by the binding of annexin V and the permeability of the plasma membrane to the 7AAD dye is a clear indicator of apoptosis in the cell. Multivariate analysis carried out using three dyes—annexin V Alexa fluor 488, 7AAD and MitoSense Red—makes it possible to clearly establish the event of apoptosis depending on damage to the mitochondrial membrane and dysfunction of mitochondria.

The change of potential (ΔΨ) in Jurkat cells after treatment with compound **13c** was assessed using the fluorescent cationic dye MitoSense Red, which selectively accumulates only in intact mitochondria of living cells and does not penetrate into dead objects.

**Figure 7:**
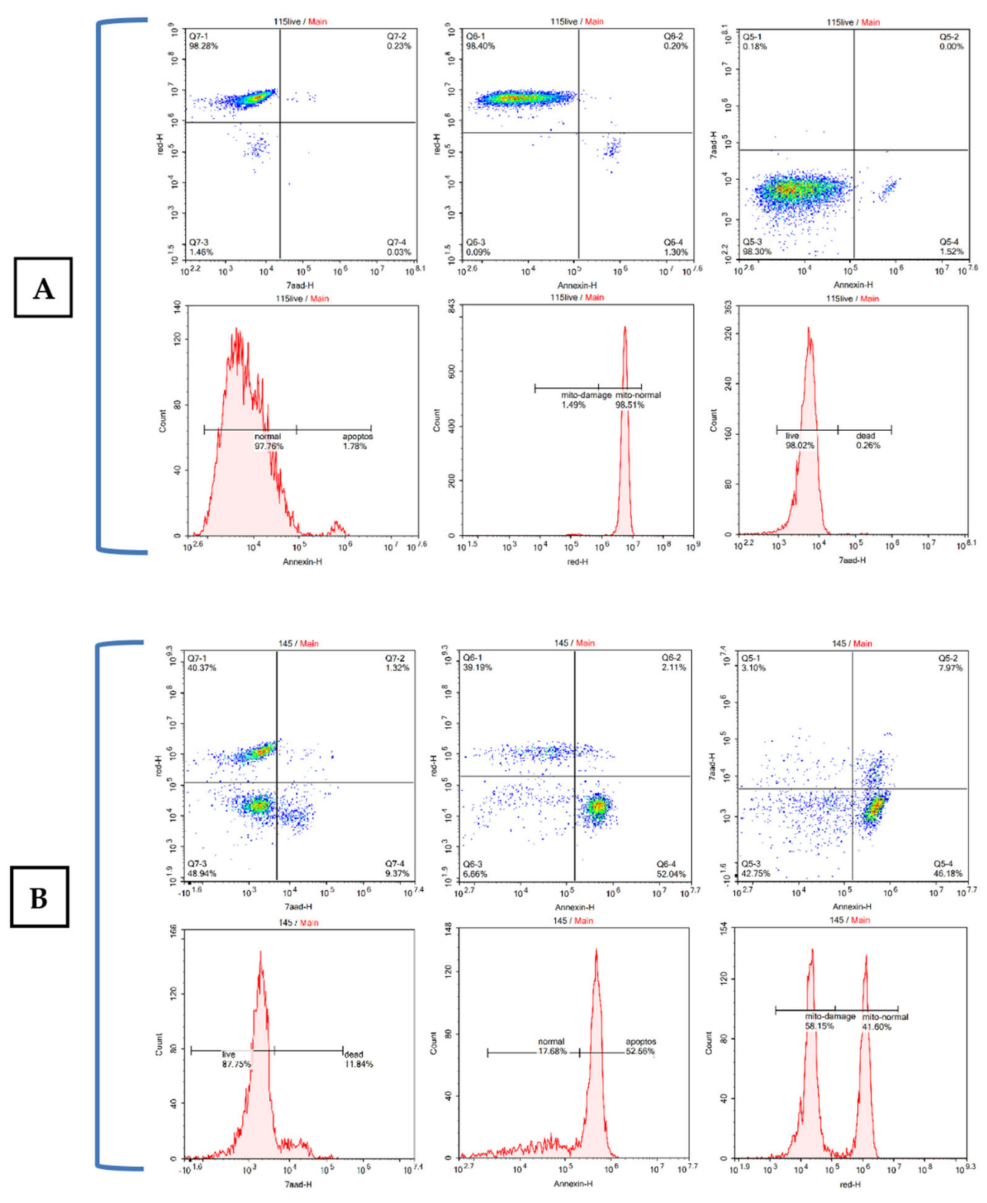

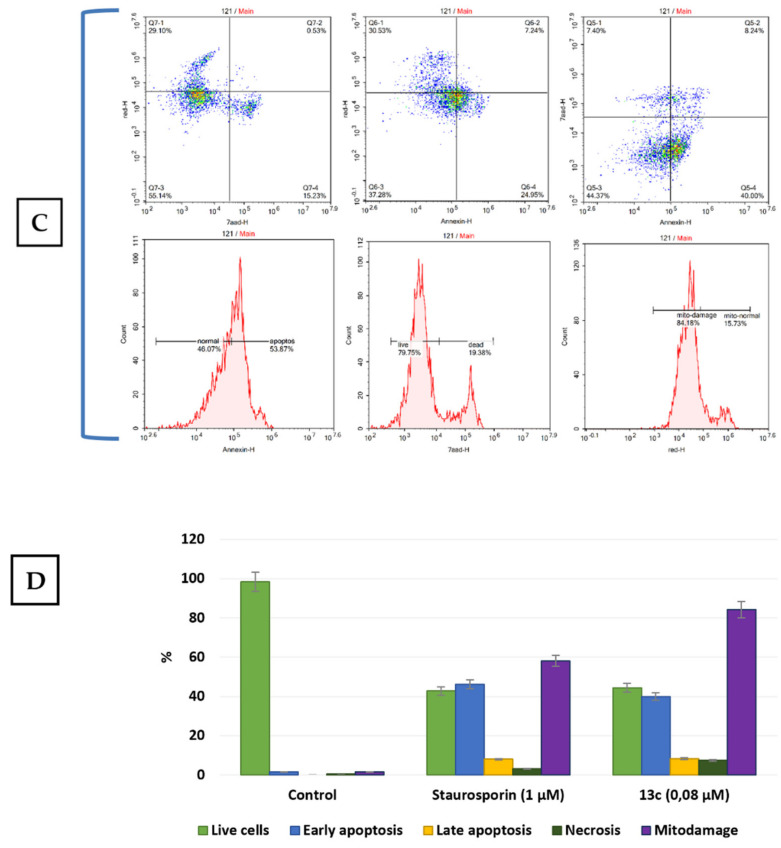
Changes in the mitochondrial potential (ΔΨ) and related early and late apoptosis in Jurkat cells induced by compound **13c**. (**A**) Control sample containing live cells; (**B**) Staurosporine (1 µM); (**C**) **13c** (0.08 µM); (**D**) Histogram showing the percentage of damaged mitochondria and phases of apoptosis. The cells were stained by MitoSense Red, Anexin V, CF488A and 7-AAD. The incubation time was 4 h. Results are expressed as mean ± SE (*n* = 3).

As a result of the experiment, it was found that in the samples treated with compound **13c**, the number of cells with damaged membrane potential (ΔΨ) increased dramatically (84.18%), while when treated with staurosporin, this number of cells was 58.15% (Figure 7, histograms B and C). Out of all trienoic acids, compound **13c** induced the most pronounced decrease in the mitochondrial potential; moreover, this effect from compound **13c** was dose-dependent and significantly exceeded the values of cells treated with staurosporin, a well-known inhibitor of protein kinases (Figure 7, histogram B). The percentage of apoptotic cells in the samples treated with compound **13c** (0.079 μM) was 48.24% (40.00% early apoptosis and 8.24% late apoptosis, respectively) (Figure 7, histogram C). The results indicate that compound 13c activates the mitochondrial pathway of apoptosis via disjunction of oxidation and phosphorylation pathways in the mitochondria of Jurkat cells.

#### 2.2.5. Cytochrome C Release from Mitochondria

An important role of mitochondria in apoptosis is associated with the release of the transmembrane protein cytochrome C, which is an activator of procaspase-9 [34]. Cytochrome c is a soluble heme protein that carries electrons in oxidative phosphorylation during respiration and transfers electrons from the cytochrome bc1 complex to cytochrome oxidase on the surface of the inner mitochondrial membrane [35]. Dissipative changes in the transmembrane potential of mitochondria (ΔΨm) indicate the appearance of signs of apoptosis in the cell [36]. Flow cytometry using detection of mitochondrial potential dissipation and cytochrome release into the cytoplasm is the method of choice for this analysis and proves the mitochondrial pathway of apoptosis in cells [37].

A quantitative assessment of the release of cytochrome c from the mitochondria of cells with apoptosis was used to detect the mitochondria-dependent pathway of cell death using flow cytometry. We used labeled antibodies against cytochrome c FITC, control of the isotype anti-IgG1-FITC, as well as optimized fixation, permeabilization and blocking buffer, allowing the detection of cytochrome c by flow cytometry. In the work, buffers were used to achieve selective mitochondrial permeability, while simultaneously leaving the mitochondrial membrane intact. Viable or living cells show higher levels of cytochrome c fluorescence, while apoptotic cells that have released their cytochrome c from the mitochondria into the cytoplasm will show a reduced staining intensity when probed with an anti-cytochrome c antibody FITC.

**Figure 8:**
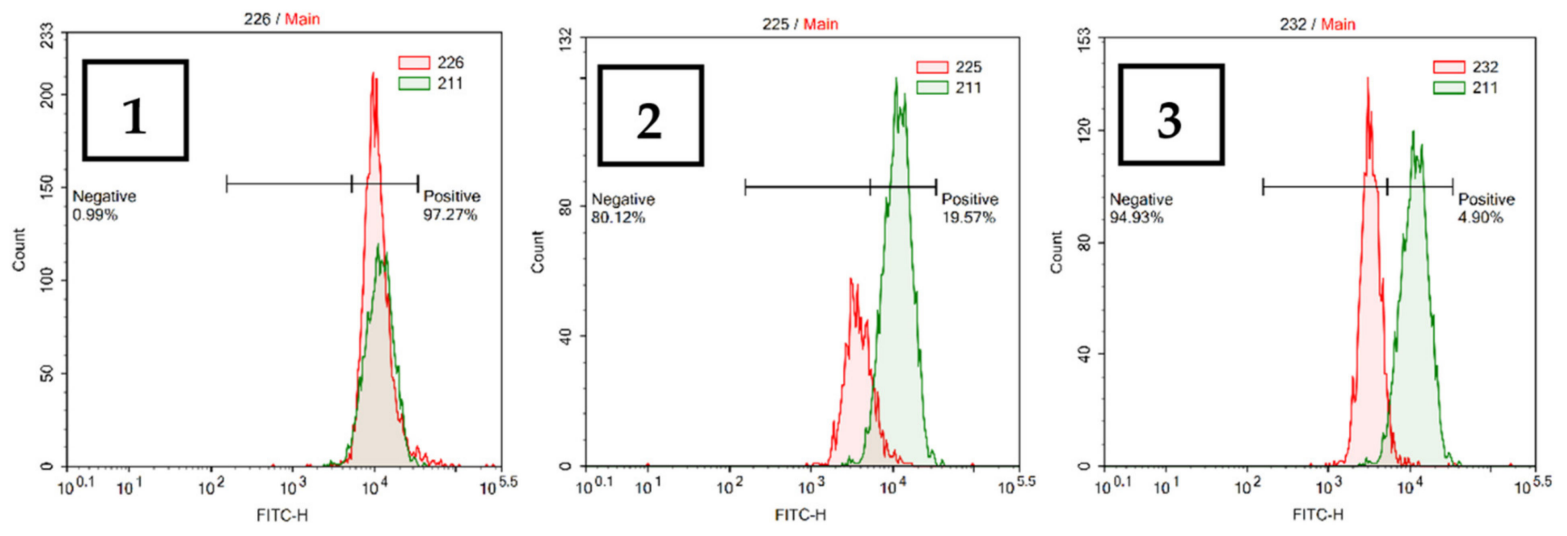
Loss of cytochrome c from the cells after incubation with trienoic acid **13c**. (**1**) untreated Jurkat cells; (**2**) cells treated with staurosporine (0.1 µM); (**3**) cells treated with compound **13c**. The incubation time was 2 h.

The detection of cytochrome c-negative cells in samples incubated with the test compound directly attests to the loss of cytochrome c with mitochondria and, as a consequence, to the intrinsic pathway of apoptosis. The histograms depicted in Figure 8 show the downward changes for the cytochrome c fluorescence for the cells incubated with test compound **13c** in a concentration of 0.08 µM. The assay was carried out using the FlowCellect Cytochrome c Kit for flow cytofluorimetry. The histograms show live cells (negative control) stained with cytochrome c-FITC and demonstrating a high level of fluorescence (Figure 8, histograms 1). These cells had intact mitochondria. In the cells that show reduced fluorescence, the membrane has been damaged by the test compound, resulting in the loss of cytochrome c. Comparison of the effects of staurosporine and the test compound **13c** shows that in 2 h, compound **13c** induces the loss of 94.93% of cytochrome c from all cells, while in the case of staurosporine, only 80% of cytochrome c is lost (Figure 8, histograms 2, 3).

#### 2.2.6. Major Kinases and Their Phosphorylation Status of Nine Signaling Pathways for Cell Growth and Proliferation

Nine major kinases of signaling pathways responsible for cell growth and differentiation were studied in Jurkat tumor cells incubated with novel trienoic acid **13c**. The study was carried out using multiplex analysis using Luminex xMAP technology. This technology makes it possible to simultaneously detect in the same experiment a number of protein analytes per unit of time, which significantly reduces the probability of errors associated with sample preparation. This method is based on the use of special polystyrene spheres coated with antibodies with special fluorophores, followed by laser detection and digital processing. In our experiment, the following kinases were analyzed: CREB, JNK, NFkB, p38, ERK1/2, Akt, p70S6K, STAT3 and STAT5, more precisely, their phosphorylated and non-phosphorylated forms in cells. Often, in many tumors, it is the activation of these kinases, all at once or only some, that gives the cell immortality, completely excluding the Hayflick limit.

**Figure 9:**
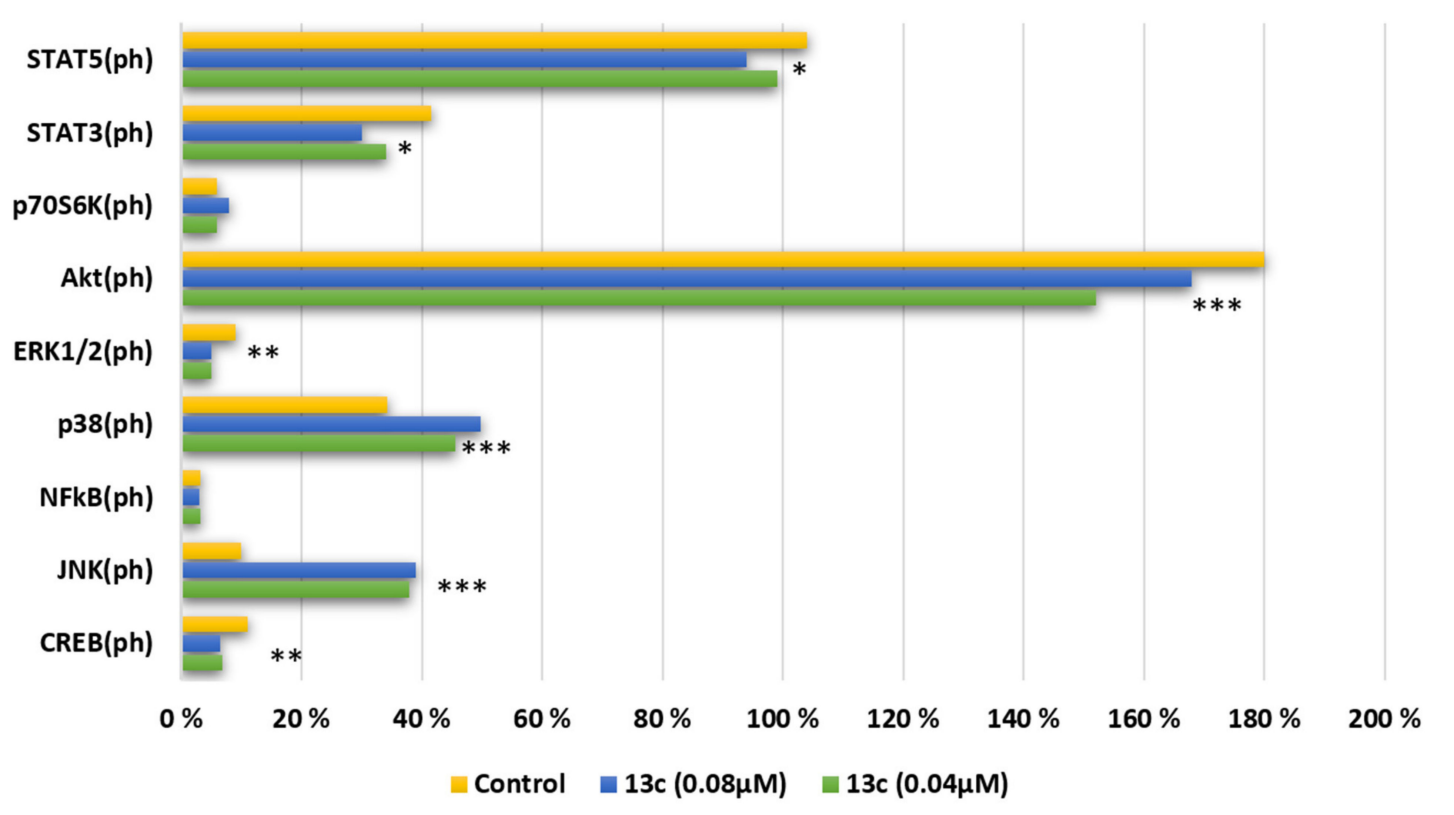
Effect of compound **13c** on intracellular signaling (CREB, JNK, NFkB, p38, ERK1/2, Akt, p70S6K, STAT3) through the activation of tyrosine kinases of the main pathways of cell proliferation and differentiation. Phosphorylation levels of key members of the 9 most important signaling pathways were determined using the MILLIPLEX^®^ MAP 9-Plex Multi-Pathway 9-plex Magnetic Bead Kit (Luminex) in protein harvested from control cells and cells treated with the substance after 24 h of incubation (Jurkat cells were used). Phosphorylation levels in control HeLa cells were taken as 100%. Phosphorylation levels were adjusted for protein load by the levels of total protein (non-phosphorylated) determined using the 9-Plex Multi-Pathway Total Magnetic Bead Kit (Luminex) in the same sample. Data are presented as mean plus standard error (* *p* > 0.05, ** *p* > 0.01, *** *p* > 0.001).

We performed a pairwise comparison of all nine kinases CREB, JNK, NFkB, p38, ERK1/2, Akt, p70S6K, STAT3 and STAT5 in an inactive and phosphorylated state in three samples of Jurkat cell lysate (samples containing 0.04 μM and 0.08 μM test compound and control). All of these samples have been tested using Luminex Assay (MILLIPLEX^®^ MAP 9-Plex Multi-Pathway 9-plex Magnetic Bead Kit) (Figure 9).

Pairwise comparison of the active and inactive forms of the protein shows that the most significant changes are detected for two signaling pathways, namely Akt and p38 (Figure 9). Kinases ERK/MAP and Akt are two key families of serine-threonine kinases Ser that are activated by RTK signals and, in turn, activate the kinases p70S6, Msk1, STAT3 (Ser727) and CREB. In addition, the simultaneous activation of FAS receptors or other stressful stimuli generated in response to intracellular signaling can cause the activation of p38, JNK and NF-kB. Figure 9 shows that compound 13c significantly reduces all types of kinase proteins at a concentration of 0.08 μM compared to the control sample. Obviously, a slight increase in the phosphorylated form of p38 is a response to stress exposure of the test compound. Akt kinase is a key enzyme of the PI3K/Akt signaling pathway involved in cell growth and differentiation [38]. In recent years, this kinase has been attributed an important role as a pro-oncogene and a participant in oncogenic cell transformation [39]. In Figure 9, it is plainly seen that the phosphorylated Akt kinase fraction considerably decreases, with this decrease being dose-dependent, in comparison with the concentration of this kinase in the control sample. Akt is an important component of phosphatidylinositol-3-kinase (PI3K) signaling pathway; it has numerous substrates and promotes signal transduction along this pathway. The PI3K and Akt kinases are important targets for the therapeutic action [40].

P38 is a MAPK kinase, that is, a serine threonine protein kinase activated by numerous external stimuli towards signal transduction from the membrane surface to the nucleus. The MAPK signaling pathway kinases are the central components of the Ras/ERK/MAPK cascade responsible for differentiation and cell growth as well. The Ras and Raf proteins are important prognostic markers of tumor diseases and promising targets for the therapy [41].

## 3. Materials and Methods

### 3.1. Chemistry

IR spectra were recorded on Bruker VERTEX 70V using KBr discs over the range of 400–4000 cm^−1^. ^1^H and ^13^C NMR spectra were obtained using a Bruker Ascend 500 spectrometer in CDCl_3_ operating at 500 MHz for ^1^H and 125 MHz for ^13^C and Bruker AVANCE 400 spectrometer in CDCl_3_ operating at 400 MHz for ^1^H and 100 MHz for ^13^C. High resolution mass spectra (HRMS) were measured on a Bruker maXis instrument using electrospray ionization (ESI). In experiments on selective collisional activation (CAD) activation energy was set at maximum abundance of fragment peaks (see figures legend). A syringe injection was used for solutions in MeCN-H_2_O, 50/50 vol.% (flow rate 3 mL/min). Nitrogen was applied as a dry gas; interface temperature was set at 180 °C. Elemental analyses were measured on a 1106 Carlo Erba apparatus. The purity of the synthesized compounds was controlled using TLC on Sorbfil plates; anisic aldehyde in AcOH was used for color development. Column chromatography was carried out on Acrus silica gel (0.060–0.200 mm). All solvents were dried (1,4-dioxane, THF, Et_2_O over Na) and freshly distilled before use.

### 3.2. Cell Culturing

Cell culturing were carried out following the known procedure [42]. Cells (Jurkat, K562, U937, HL60, HeLa, HEK293 and normal fibroblasts) were purchased from HPA Culture Collections (Salisbury, UK) and cultured according to standard mammalian tissue culture protocols and sterile technique.

### 3.3. DNA Topoisomerase I Assay

Inhibition of DNA topoisomerase I tests were carried out following the known procedure [25]. Human topoisomerase I inhibition studies were carried out using commercially available Topoisomerase I Drug Screening Kit (TG-1018-2, Topogen, CO, USA).

### 3.4. Cytotoxicity Assay

Cytotoxicity tests were carried out following the known procedure [42]. Viability (live/dead) assessment was performed by staining cells with 7-AAD (7-Aminoactinomycin D) (Biolegend). After treatment cells were harvested, washed 1–2 times with phosphate-buffered saline (PBS) and centrifuged at 400 g for 5 min. Cell pellets were resuspended in 200 mL of flow cytometry staining buffer (PBS without Ca^2+^ and Mg^2+^, 2.5% FBS) and stained with 5 μL of 7-AAD staining solution for 15 min at room temperature in the dark. Samples were acquired on NovoCyteTM 2000 FlowCytometry System (ACEA) equipped with 488 nm argon laser. Detection of 7-AAD emission was collected through a 675/30 nm filter in FL4 channel.

### 3.5. Viability and Apoptosis

Induction of apoptosis tests were carried out following the known procedure [42]. Apoptosis was determined by flow cytometric analysis of Annexin V and 7-aminoactinomycin D staining. After treatment cells during 24 h were harvested, washed 1–2 times with phosphate-buffered saline (PBS) and centrifuged at 400 g for 5 min. Cell pellets were resuspended in 200 μL of flow cytometry staining buffer (PBS without Ca^2+^and Mg^2+^, 2.5% FBS). Then, 200 μL of Guava Nexin reagent (Millipore, Bedford, MA, USA) was added to 5 × 10^5^ cells in 200 μL and the cells were incubated with the reagent for 20 min at room temperature in the dark. At the end of incubation, the cells were analyzed on NovoCyteTM2000 FlowCytometry System (ACEA).

### 3.6. Cell Cycle Analysis

Cell cycle analysis was carried out following the known procedure [42]. Guava Cell Cycle Reagent (Millipore) was used. Samples were analyzed on NovoCyteTM 2000FlowCytometry System (ACEA).

### 3.7. Mitochondrial Damage

Mitochondrial damage tests were carried out following the known procedure [42]. Cytometric assay (Millipore’s FlowCellect™MitoDamage Kit) which allowed multiparametric evaluation of three cell health markers: change in mitochondrial potential (early apoptosis and cellular stress), phosphatidylserine expression on the cell surface (late apoptosis) and membrane permeabilization (cell death) was used.

### 3.8. Histone H2A.X Analysis

Histone H2A.X analysis was carried out following the known procedure [42]. Cell preparation for phosphorylation of Histone H2A.X in Jurkat cells analysis were grown in culture flask at 48 h. The medium was changed at 24 h before drug treatment. Phosphorylation of histone H2A.X was measured with the FlowCellect™ DNA Damage Histone H2A.X Dual Detection Kit (FCCS025153, Millipore, MA, USA).

### 3.9. Cytochrome C Release Analysis

The commercially available Millipore FlowCellect ™ Cytochrome c kit measures the loss of mitochondrial cytochrome c in cells in which apoptosis has been induced. The kit includes a directly labeled anti-cytochrome cFITC antibody, an anti-IgG1-FITC isotype control, as well as buffers for fixation and permeabilization of cells, allowing the detection of cytochrome by flow cytometry. Viable or living cells show higher levels of fluorescence of cytochrome c and the mitochondria themselves, while apoptotic cells that have released their cytochrome c from the mitochondria into the cytoplasm will show a reduced intensity of staining when probed with an anti-cytochrome c FITC antibody. The incubation time of the cells with the test substances was 4 h.

### 3.10. Multiplex Analysis of Early Apoptosis Markers

Multiplex analysis was carried out following the known procedure [42]. The MILLIPLEX^®^ MAP 9-Plex Multi-Pathway Kits, total, are used to detect total protein levels or phosphorylated protein levels for ERK/MAP kinase 1/2, Akt, STAT3, JNK, p70 S6 kinase, NFκB, STAT5A/B, CREB and p38 in cell lysates using the Luminex^®^ system. The detection assay is a rapid, convenient alternative to Western Blotting and immunoprecipitation procedures. Each kit has sufficient reagents for one 96 well plate assay. Multiplexed immunoassays 9-Plex Multi-PathwayTotal Magnetic Bead Kit 96-well Platefrom Millipore were used to detect changes in phosphorylated cAMP response element-binding protein (CREB; pS133), ERK (pT185/pY187), NF-kB (pS536), JNK (pT183/pY185), p38 (pT180/pY182), p70 S6K (pT412), STAT3 (pS727), STAT5A/B (pY694/699) and Akt (pS473) (Milliplex 48-680MAG; Merck Millipore, Germany) in Jurkat cell lysates lysed with RIPA buffer and protease and phosphatase inhibitors according to the array protocol. Before lysis in Milliplex MAP Lysis Buffer, the cells were challenged for 3 min with 100 ng/mL EGF and 10 nM HRG-b1 and prepared for subsequent analysis.

### 3.11. Chemical Experimental Data

#### 3.11.1. Procedure for Preparation of Alka-3-in-1-ols (**4a**–**e**)

To a solution of alk-1-yne (80 mmol) in THF (50 mL) was slowly added ethyl magnesium bromide (3.0 M in THF, 32.0 mL, 96.0 mmol). The mixture was heated under reflux for a further 4 h then cooled over ice. Ethylene oxide (5.85 g, 130 mmol) was decanted into ice cold dry THF (30 mL) and slowly added to the cooled Grignard reagent. A slight exotherm was observed on this addition. The mixture was allowed to warm to room temperature then stirred overnight prior to cooling over ice then hydrolyzed by careful addition of HCl (50 mL 5%). The mixture was extracted with EtOAc (2 × 100 mL), dried over MgSO_4_ and evaporated to give **4a**–**e** as a colorless oil.

##### Oct-3-yn-1-ol (**4a**)

88% yield. ^1^H NMR (CDCl_3_, 400 MHz) *δ* 3.67 (2H, t, *J* = 6.4 Hz), 2.44–2.42 (2H, m), 2.18–2.11 (2H, m), 1.49–1.39 (4H, m), 0.91 (3H, t, *J* = 6.7 Hz); ^13^C NMR (CDCl_3_, 100.62 MHz) *δ* 82.65 (C-4), 76.26 (C-3), 61.37 (C-1), 31.06 (C-6), 23.15 (C-2), 21.94 (C-7), 18.40 (C-5), 13.57 (C-8); anal. calcd for C_8_H_14_O: C, 76.13; H, 11.18; found: C, 76.09; H, 11.11; HRMS (ESI-TOF): calcd for C_8_H_14_O [M + H]^+^127.2041; found 127.2038.

##### Dec-3-yn-1-ol (**4b**)

85% yield. ^1^H NMR (CDCl_3_, 400 MHz) *δ* 3.66 (2H, t, *J* = 6.4 Hz), 2.44–2.40 (2H, m), 2.18–2.13 (2H, m), 1.52–1.24 (8H, m), 0.88 (3H, t, *J* = 6.7 Hz); ^13^C NMR (CDCl_3_, 100.62 MHz) *δ* 82.65 (C-4), 76.27 (C-3), 61.35 (C-1), 31.33 (C-8), 28.94 (C-7), 28.54 (C-6), 23.13 (C-2), 22.52 (C-9), 18.71 (C-5), 13.99 (C-10); anal. calcd for C_10_H_18_O: C, 77.86; H, 11.76; found: C, 77.69; H, 11.63; HRMS (ESI-TOF): calcd for C_10_H_18_O [M + H]^+^155.2572; found 155.2568.

##### Dodec-3-yn-1-ol (**4c**)

79% yield. ^1^H NMR (CDCl_3_, 400 MHz) *δ* 3.59 (2H, t, *J* = 6.4 Hz), 2.36–2.32 (2H, m), 2.10–2.06 (2H, m), 1.46–1.22 (12H, m), 0.82 (3H, t, *J* = 6.7 Hz); ^13^C NMR (CDCl_3_, 100.62 MHz) *δ* 82.14 (C-4), 76.34 (C-3), 61.23 (C-1), 31.79 (C-10), 29.15 (C-7), 29.07 (C-9), 28.96 (C-8), 28.84 (C-6), 22.98 (C-2), 22.58 (C-11), 18.65 (C-5), 13.95 (C-12); anal. calcd for C_12_H_22_O: C, 79.06; H, 12.16; found: C, 78.91; H, 12.02; HRMS (ESI-TOF): calcd for C_12_H_22_O [M + H]^+^183.3104; found 183.3100.

##### Tetradec-3-yn-1-ol (**4d**)

83% yield. ^1^H NMR (CDCl_3_, 400 MHz) *δ* 3.68 (2H, t, *J* = 6.4 Hz), 2.46–2.41 (2H, m), 2.21–2.14 (2H, m), 1.53–1.27 (16H, m), 0.88 (3H, t, *J* = 6.7 Hz); ^13^C NMR (CDCl_3_, 100.62 MHz) *δ* 82.72 (C-4), 76.25 (C-3), 61.37 (C-1), 31.89 (C-12), 29.57 (C-8), 29.52 (C-10), 29.30 (C-9), 29.14 (C-11), 29.00 (C-8), 28.89 (C-6), 23.17 (C-2), 22.66 (C-13), 18.72 (C-5), 14.06 (C-14); anal. calcd for C_14_H_26_O: C, 79.93; H, 12.45; found: C, 79.85; H, 12.33; HRMS (ESI-TOF): calcd for C_14_H_26_O [M + H]^+^211.3635, found 211.3630.

##### Hexadec-3-yn-1-ol (**4e**)

89% yield. ^1^H NMR (CDCl_3_, 400 MHz) *δ* 3.68 (2H, t, *J* = 6.4Hz), 2.45–2.40 (2H, m), 2.18–2.13 (2H, m), 1.52–1.27 (20H, m), 0.88 (3H, t, *J* = 6.7 Hz); ^13^C NMR (CDCl_3_, 100.62 MHz) *δ* 82.71 (C-4), 76.26 (C-3), 61.36 (C-1), 31.92 (C-14), 29.67 (C-9), 29.64 (C-7, C-11), 29.54 (C-12), 29.35 (C-13), 29.16 (C-10), 29.00 (C-8), 28.91 (C-6), 23.16 (C-2), 22.68 (C-15), 18.73 (C-5), 14.09 (C-16); anal. calcd for C_16_H_30_O: C, 80.60; H, 12.68; found: C, 80.52; H, 12.60; HRMS (ESI-TOF): calcd for C_16_H_30_O [M + H]^+^239.4167, found 239.4161.

#### 3.11.2. Synthesis of (Z)-alk-3-en-1-ols (**5**)

To a stirred mixture of Ni(OAc)_2_ × 4H_2_O (10.7 g, 42.7 mmol) in ethanol (86 mL) was added NaBH_4_ (1.2 g, 31 mmol) in ethanol (43 mL) at room temperature under hydrogen. The mixture was stirred for 60 min and ethylenediamine (10.32 g, 171.5 mmol) and alkynol **4a**–**e** (43 mmol) were added sequentially. After stirring for 12 h at the same temperature under hydrogen, the reaction mixture was concentrated and diluted with ethyl acetate. The resulting mixture was filtered through a celite pad and the filtrate was concentrated under reduced pressure. The residue was purified by silica gel chromatography (n-hexane/ethyl acetate 5:1) to furnish **5a**–**e** as a colorless oil.

##### *(Z)*-Oct-3-en-1-ol (**5a**)

96% yield. ^1^H NMR (CDCl_3_, 400 MHz) *δ* 5.57–5.26 (2H, m), 3.68 (2H, dt, *J* = 6.6 Hz), 2.37–2.29 (2H, m), 2.08–2.02 (2H, m), 1.35–1.20 (4H, m), 0.89 (3H, t, *J* = 6.7 Hz); ^13^C NMR (CDCl_3_, 100.62 MHz) *δ* 133.23 (C-4), 124.97 (C-3), 62.22 (C-1), 31.84 (C-6), 30.72 (C-2), 27.03 (C-5), 22.31 (C-7), 13.90 (C-8); anal. calcd for C_8_H_16_O: C, 74.94; H, 12.57; found: C, 74.87; H, 12.49; HRMS (ESI-TOF): calcd for C_8_H_16_O [M + H]^+^129.2199, found 129.2192.

##### *(Z)-*Dec-3-en-1-ol (**5b**)

91% yield. ^1^H NMR (CDCl_3_, 400 MHz) *δ* 5.62–5.32 (2H, m), 3.67 (2H, t, *J* = 6.6 Hz), 2.38–2.33 (2H, m), 2.11–2.06 (2H, m), 1.37–1.28 (8H, m), 0.90 (3H, t, *J* = 6.7 Hz); ^13^C NMR (CDCl_3_, 100.62 MHz) *δ* 133.63 (C-4), 124.91 (C-3), 62.37 (C-1), 31.89 (C-8), 30.81 (C-2), 29.71 (C-7), 29.32 (C-6), 27.39 (C-5), 22.68 (C-9), 14.11 (C-10); anal. calcd for C_10_H_20_O: C, 76.86; H, 12.90; found: C, 76.80; H, 12.82; HRMS (ESI-TOF): calcd for C_10_H_20_O [M + H]^+^157.2714, found 157.2710.

##### *(Z)-*Dodec-3-en-1-ol (**5c**)

94% yield. ^1^H NMR (CDCl_3_, 400 MHz) *δ* 5.62–5.32 (2H, m), 3.66 (2H, t, *J* = 6.6 Hz), 2.38–2.33 (2H, m), 2.11–2.06 (2H, m), 1.43–1.26 (12H, m), 0.90 (3H, t, *J* = 6.7 Hz); ^13^C NMR (CDCl_3_, 100.62 MHz) *δ* 133.35 (C-4), 124.96 (C-3), 62.25 (C-1), 31.91 (C-10), 30.76 (C-2), 29.71 (C-6), 29.64 (C-7), 29.55 (C-8), 29.35 (C-9), 27.37 (C-5), 22.68 (C-11), 14.09 (C-12); anal. calcd for C_12_H_24_O: C, 78.19; H, 13.12; found: C, 78.10; H, 13.02; HRMS (ESI-TOF): calcd for C_12_H_24_O [M + H]^+^185.3263, found 185.3258.

##### *(Z)-*Tetradec-3-en-1-ol (**5d**)

98% yield. ^1^H NMR (CDCl_3_, 400 MHz) *δ* 5.58–5.33 (2H, m), 3.62 (2H, t, *J* = 6.6 Hz,), 2.35–2.30 (2H, m, *J* = 6.6 Hz), 2.10–2.00 (2H, m), 1.37–1.23 (16H, m), 0.88 (3H, t, *J* = 6.7 Hz); ^13^C NMR (CDCl_3_, 100.62 MHz) *δ* 133.35 (C-4), 124.96 (C-3), 62.25 (C-1), 31.91 (C-12), 30.76 (C-2), 29.71 (C-8), 29.64 (C-6), 29.55 (C-7), 29.35 (C-9), 29.32 (C-10, C-11), 27.37 (C-5), 22.68 (C-13), 14.09 (C-14); anal. calcd for C_14_H_28_O: C, 79.17; H, 13.28; found: C, 79.09; H, 13.19; HRMS (ESI-TOF): calcd for C_14_H_28_O [M + H]^+^213.3794, found 213.3790.

##### *(Z)-*Hexadec-3-en-1-ol (**5e**)

98% yield. ^1^H NMR (CDCl_3_, 400 MHz) *δ* 5.59–5.33 (2H, m), 3.63 (2H, t, *J* = 6.6 Hz), 2.35–2.30 (2H, m), 2.09–2.04 (2H, m), 1.37–1.27 (20H, m), 0.88 (3H, t, *J* = 6.7 Hz); ^13^C NMR (CDCl_3_, 100.62 MHz) *δ* 133.38 (C-4), 124.98 (C-3), 62.27 (C-1), 31.93 (C-14), 30.81 (C-2), 29.72 (C-8), 29.69 (C-6, C-7), 29.65 (C-10, C-11), 29.56 (C-12), 29.36 (C-9), 29.33 (C-13), 27.38 (C-5), 22.69 (C-15), 14.10 (C-16); anal. calcd for C_16_H_32_O: C, 79.92; H, 13.41; found: C, 79.86; H, 13.32; HRMS (ESI-TOF): calcd for C_16_H_32_O [M + H]^+^241.4326, found 241.4320.

#### 3.11.3. Synthesis of (Z)-1-Bromoalk-3-enes (**7**)

A mixture of methane sulphonyl chloride (4.93 g, 43.0 mmol), triethylamine (8.22 mL), (Z)-alk-3-en-1-ol (**5a**–**e**) (34.0 mmol) and CH_2_Cl_2_ (52.2 mL) was stirred at 0 °C for 1 h. The reaction mixture was filtered and the residue washed with CH_2_Cl_2_ (40 mL). The combined CH_2_Cl_2_ layer was washed with dil. HCl (30 mL), water (2 × 25 mL), dried (Na_2_SO_4_) and the solvent removed under reduced pressure. The solution of the 6a–e in dry acetone (104 mL) was added LiBr (6.0 g, 69.2 mmol) and the mixture refluxed for 3 h. Ice cold water (60 mL) was added and the oil that separated out removed. The aqueous layer was extracted with n-hexane (2 × 50 mL). The n-hexane extract was combined with the oily liquid, washed with water (20 mL) and dried over anhydrous sodium sulphate. Evaporation of the solvent, followed by column chromatography of the residue on silica gel, using n-hexane-diethyl ether (95:5) as eluent, gave **7a**–**e** as a yellow liquid oil.

##### *(Z)-*1-Bromoct-3-ene (**7a**)

91% yield. ^1^H NMR (CDCl_3_, 400 MHz) *δ* 5.57–5.32 (2H, m), 3.36 (2H, t, *J* = 7.2 Hz), 2.65–2.59 (2H, m), 2.18–2.03 (2H, m), 1.38–1.24 (4H, m), 0.90 (3H, t, *J* = 6.7 Hz); ^13^C NMR (CDCl_3_, 100.62 MHz) *δ* 133.11 (C-4), 125.74 (C-3), 32.52 (C-1), 31.69 (C-2), 30.84 (C-6), 27.12 (C-5), 22.31 (C-7), 13.94 (C-8); anal. calcd for C_8_H_15_Br: C, 50.27; H, 7.99; found: C, 50.17; H, 7.88; HRMS (ESI-TOF): calcd for C_8_H_15_Br [M + H]^+^192.1087, found 192.1076.

##### *(Z)-*1-Bromdec-3-ene (**7b**)

90% yield. ^1^H NMR (CDCl_3_, 400 MHz) *δ* 5.59–5.34 (2H, m), 3.38 (2H, t, *J* = 7.2 Hz), 2.64–2.61 (4H, m), 1.39–1.28 (8H, m), 0.90 (3H, t, *J* = 6.7 Hz); ^13^C NMR (CDCl_3_, 100.62 MHz) *δ* 133.21 (C-4), 125.73 (C-3), 32.54 (C-1), 31.73 (C-8), 30.86 (C-2), 29.49 (C-7), 28.95 (C-6), 27.44 (C-5), 22.63 (C-9), 14.07 (C-10); anal. calcd for C_10_H_19_Br: C, 54.80; H, 8.73; found: C, 54.73; H, 8.66; HRMS (ESI-TOF): calcd for C_10_H_19_Br [M + H]^+^220.1698, found 220.1690.

##### *(Z)-*1-Bromdodec-3-ene (**7c**)

89% yield. ^1^H NMR (CDCl_3_, 400 MHz) *δ* 5.59–5.34 (2H, m), 3.38 (2H, t, *J* = 7.2 Hz), 2.66–2.61 (2H, m), 2.11–2.03 (2H, m), 1.38–1.26 (12H, m), 0.91 (3H, t, *J*= 6.7 Hz); ^13^C NMR (CDCl_3_, 100.62 MHz) *δ* 133.22 (C-4), 125.72 (C-3), 32.55 (C-1), 31.88 (C-10), 30.86 (C-2), 29.53 (C-7), 29.48 (C-8), 29.28 (C-6, C-9), 27.43 (C-5), 22.67 (C-11), 14.09 (C-12); anal. calcd for C_12_H_23_Br: C, 58.30; H, 9.38. Found: C, 58.22; H, 9.31; HRMS (ESI-TOF): calcd for C_12_H_23_Br [M + H]^+^248.2229, found 248.2220.

##### *(Z)-*1-Bromtetradec-3-ene (**7d**)

93% yield. ^1^H NMR (CDCl_3_, 400 MHz) *δ* 5.59–5.35 (2H, m), 3.38 (2H, t, *J* = 7.2 Hz), 2.67–2.61 (2H, m), 2.09–2.01 (2H, m), 1.45–1.29 (16H, m), 0.91 (3H, t, *J* = 6.7 Hz); ^13^C NMR (CDCl_3_, 100.62 MHz) *δ* 133.18 (C-4), 125.73 (C-3), 32.44 (C-1), 31.93 (C-12), 30.88 (C-2), 29.64 (C-6, C-7), 29.54 (C-8, C-9), 29.36 (C-10), 29.30 (C-11), 27.44 (C-5), 22.70 (C-13), 14.10 (C-14); anal. calcd for C_14_H_27_Br: C, 61.08; H, 9.88; found: C, 60.00; H, 9.79; HRMS (ESI-TOF): calcd for C_14_H_27_Br [M + H]^+^276.2761, found 276.2758.

##### *(Z)-*1-Bromhexadec-3-ene (**7e**)

95% yield. ^1^H NMR (CDCl_3_, 400 MHz) *δ* 5.59–5.35 (2H, m), 3.37 (2H, t, *J* = 7.2 Hz), 2.66–2.61 (2H, m), 2.08–2.03 (2H, m), 1.40–1.29 (20H, m), 0.90 (3H, t, *J* = 6.7 Hz); ^13^C NMR (CDCl_3_, 100.62 MHz) *δ* 133.17 (C-4), 125.73 (C-3), 32.42 (C-1), 31.97 (C-14), 30.89 (C-2), 29.73 (C-6, C-7),29.67(C-10, C-11), 29.56 (C-8, C-12), 29.41 (C-9), 29.32 (C-13), 27.45 (C-5), 22.73 (C-15), 14.13 (C-16); anal. calcd for C_16_H_31_Br: C, 63.35; H, 10.30; found: C, 63.28; H, 10.22; HRMS (ESI-TOF): calcd for C_16_H_31_Br [M + H]^+^304.3292, found 304.3287.

#### 3.11.4. Synthesis of Alk-5-en-1-ynes (**8a**–**e**)

To a suspension of a lithium acetylide-ethylenediamine complex (3.1 g, 36.9 mmol) in DMSO (13.6 mL) was added **7a**–**e** (22.2 mmol) in DMSO (11.2 mL) at 0 °C. The reaction mixture was stirred for 12 h 23 °C. After the reaction had been completed, sat. NH_4_Cl (10 mL) was added to the mixture. The mixture was extracted with ether, washed with brine, dried over MgSO_4_ and concentrated. The residue was chromatographed over silica gel (hexane) to afford 8a–e as a colorless oil.

##### *(Z)-*Dec-5-en-1-yne (**8a**)

67% yield. ^1^H NMR (CDCl_3_, 400 MHz) *δ* 5.50–5.40 (2H, m), 2.31–2.17 (6H, m), 2.07–2.03 (1H, m), 1.40–1.20 (4H, m), 0.91 (3H, t, *J* = 6.7 Hz); ^13^C NMR (CDCl_3_, 100.62 MHz) *δ* 131.59 (C-6), 129.11 (C-5), 84.18 (C-2), 68.25 (C-1), 31.75 (C-8), 27.41 (C-7), 26.99 (C-4), 22.27 (C-9), 18.84 (C-3), 13.89 (C-10); anal. calcd for C_10_H_16_: C, 88.16; H, 11.83; found: C, 88.10; H, 11.78; HRMS (ESI-TOF): calcd for C_10_H_16_ [M + H]^+^137.2419, found 137.2412.

##### *(Z)-*Dodec-5-en-1-yne (**8b**)

68% yield. ^1^H NMR (CDCl_3_, 400 MHz) *δ* 5.52–5.09 (2H, m), 2.32–2.18 (6H, m), 2.11–2.04 (1H, m), 1.45–1.33 (10H, m), 0.91 (3H, t, *J* = 6.7 Hz); ^13^C NMR (CDCl_3_, 100.62 MHz) *δ* 133.09 (C-6), 129.10 (C-5), 84.27 (C-2), 68.25 (C-1), 31.73 (C-10), 29.59 (C-9), 28.92 (C-8), 27.75 (C-7), 27.32 (C-4), 22.63 (C-11), 18.86 (C-3), 14.08 (C-12); anal. calcd for C_12_H_22_: C, 86.66; H, 13.33; found: C, 86.59; H, 13.25; HRMS (ESI-TOF): calcd for C_12_H_22_ [M + H]^+^167.3110, found 167.3107.

##### *(Z)-*Tetradec-5-en-1-yne (**8c**)

72% yield. ^1^H NMR (CDCl_3_, 400 MHz) *δ* 5.52–5.09 (2H, m), 2.32–2.18 (6H, m), 2.10–2.04 (1H, m), 1.43–1.29 (12H, m), 0.91 (3H, t, *J* = 6.7 Hz); ^13^C NMR (CDCl_3_, 100.62 MHz) *δ* 133.04 (C-6), 129.12 (C-5), 84.19 (C-2), 68.25 (C-1), 31.90 (C-12), 29.64 (C-9, C-10),29.49 (C-8), 29.27 (C-11), 27.75 (C-7), 27.32 (C-4), 22.63 (C-3), 18.86 (C-13), 14.09 (C-14); anal. calcd for C_14_H_24_: C, 87.42; H, 12.57; found: C, 87.38; H, 12.49; HRMS (ESI-TOF): calcd for C_14_H_24_ [M + H]^+^193.3483, found 193.3479.

##### *(Z)-*Hexadec-5-en-1-yne (**8d**)

69% yield. ^1^H NMR (CDCl_3_, 400 MHz) *δ* 5.51–5.09 (2H, m), 2.33–2.19 (6H, m), 2.09–2.05 (1H, m), 1.47–1.30 (16H, m), 0.92 (3H, t, *J* = 6.7 Hz); ^13^C NMR (CDCl_3_, 100.62 MHz) *δ* 132.34 (C-6), 129.14 (C-5), 84.08 (C-2), 68.22 (C-1), 31.94 (C-14), 29.70 (C-10), 29.65 (C-9, C-11), 29.54 (C-8), 29.38 (C-12), 29.28 (C-13), 27.74 (C-7), 27.32 (C-4), 22.71 (C-3), 18.86 (C-15), 14.08 (C-16); anal. calcd for C_16_H_28_: C, 87.19; H, 12.80; found: C, 87.11; H, 12.73; HRMS (ESI-TOF): calcd for C_16_H_28_ [M + H]^+^221.4014, found 221.4009.

##### *(Z)-*Octadec-5-en-1-yne (**8e**)

70% yield. ^1^H NMR (CDCl_3_, 400 MHz) *δ* 5.51–5.09 (2H, m), 2.32–2.20 (6H, m), 2.10–2.06 (1H, m), 1.48–1.32 (20H, m), 0.92 (3H, t, *J* = 6.7 Hz); ^13^C NMR (CDCl_3_, 100.62 MHz) *δ* 132.31 (C-6), 129.21 (C-5), 83.92 (C-2), 68.21 (C-1), 32.01 (C-16), 29.75 (C-10, C-12, C-9), 29.71 (C-13), 29.60 (C-14), 29.46 (C-8), 29.38 (C-11), 29.32 (C-15), 27.76 (C-7), 27.35 (C-4), 22.75 (C-17), 18.87 (C-3), 14.09 (C-18); anal. calcd for C_18_H_32_: C, 87.01; H, 12.98; found: C, 86.97; H, 12.88; HRMS (ESI-TOF): calcd for C_18_H_32_ [M + H]^+^249.4546, found 249.4540.

#### 3.11.5. Synthesis of (Z)-alk-1,2,6-trienes (**9a**–**e**)

Paraformaldehyde (1.0 g), copper iodide (1.26 g, 6.8 mmol) and dicyclohexylamine (75.7 mL) were sequentially added to a solution of **8a**–**e** (16.4 mmol) in anhydrous dioxane (75.7 mL). The resulting mixture was heated under reflux for 8 h. The solvent was evaporated in vacuo. The addition of 2 M HCl (50 mL) and extraction with diethyl ether was followed by an extraction of the organic layer with NaHCO_3_, water and brine and a drying with anhydrous MgSO_4_ and the residue was purified by silica gel column chromatography (hexane/ethyl acetate = 30/1) to afford **9a**–**e** as colorless liquid.

##### *(Z)-*Undeca-1,2,6-triene (**9a**)

76% yield. ^1^H NMR (CDCl_3_, 400 MHz) *δ* 5.44–5.32 (2H, m), 5.15–5.12 (1H, m), 4.71–4.67 (2H, m), 2.20–2.04 (6H, m), 1.38–1.33 (4H, m), 0.92 (3H, t, *J* = 6.7 Hz); ^13^C NMR (CDCl_3_, 100.62 MHz) *δ* 208.50 (C-2), 130.71 (C-7), 128.65 (C-6), 89.65 (C-3), 74.82 (C-1), 31.90 (C-9), 28.41 (C-5), 26.99 (C-8), 26.84 (C-4), 22.34 (C-10), 13.99 (C-11); anal. calcd for C_11_H_18_: C, 87.92; H, 12.07; found: C, 87.87; H, 11.98; HRMS (ESI-TOF): calcd for C_11_H_18_ [M + H]^+^151.2685, found 151.2680.

##### *(Z)-*Trideca-1,2,6-triene (**9b**)

72% yield. ^1^H NMR (CDCl_3_, 400 MHz) *δ* 5.49–5.21 (m, 2H), 5.15–5.08 (1H, m), 4.71–4.67 (2H, m), 2.23–2.05 (6H, m), 1.45–1.29 (8H, m), 0.89 (3H, t, *J* = 6.7 Hz); ^13^C NMR (CDCl_3_, 100.62 MHz) *δ* 205.56 (C-2), 133.08 (C-7), 129.10 (C-6), 89.64 (C-3), 74.79 (C-1), 31.72 (C-11), 29.67 (C-5), 29.58 (C-9), 28.91 (C-10), 27.74 (C-8), 27.29 (C-4), 22.61 (C-12), 14.07 (C-13); anal. calcd for C_13_H_22_: C, 87.56; H, 12.43; found: C, 87.48; H, 12.37; HRMS (ESI-TOF): calcd for C_13_H_22_ [M + H]^+^179.3217, found 179.3211.

##### *(Z)*-Pentadeca-1,2,6-triene (**9c**)

74% yield. ^1^H NMR (CDCl_3_, 400 MHz) *δ* 5.51–5.18 (2H, m), 5.17–5.08 (1H, m), 4.71–4.68 (2H, m), 2.24–2.03 (6H, m), 1.45–1.29 (12H, m), 0.91 (3H, t, *J* = 6.7 Hz); ^13^C NMR (CDCl_3_, 100.62 MHz) *δ* 204.19 (C-2), 133.09 (C-7), 129.10 (C-6), 89.64 (C-3), 74.79 (C-1), 31.89 (C-13), 29.62 (C-5), 29.47 (C-9), 29.32 (C-10), 29.28 (C-11), 29.25 (C-12), 27.74 (C-8), 27.29 (C-4), 22.67 (C-14), 14.09 (C-15); anal. calcd for C_15_H_26_: C, 87.30; H, 12.69; found: C, 87.24; H, 12.58; HRMS (ESI-TOF): calcd for C_15_H_26_ [M + H]^+^207.3748, found 207.3742.

##### *(Z)-*Heptadeca-1,2,6-triene (**9d**)

77% yield. ^1^H NMR (CDCl_3_, 400 MHz) *δ* 5.50–5.38 (2H, m), 5.22–5.09 (1H, m), 4.71–4.68 (2H, m), 2.24–2.04 (6H, m), 1.46–1.30 (16H, m), 0.92 (3H, t, *J* = 6.7 Hz); ^13^C NMR (CDCl_3_, 100.62 MHz) *δ* 208.54 (C-2), 133.01 (C-7), 129.12 (C-6), 89.62 (C-3), 74.75 (C-1), 31.93 (C-15), 29.72 (C-5), 29.63 (C-11), 29.58 (C-9), 29.52 (C-12), 29.35 (C-13), 29.33 (C-14), 29.26 (C-10), 27.74 (C-8), 27.29 (C-4), 22.69 (C-16), 14.08 (C-17); anal. calcd for C_17_H_30_: C, 87.10; H, 12.89. Found: C, 87.03; H, 12.80; HRMS (ESI-TOF): calcd for C_17_H_30_ [M + H]^+^235.4280, found 235.4273.

##### *(Z)-*Nonadeca-1,2,6-triene (**9e**)

75% yield. ^1^H NMR (CDCl_3_, 400 MHz) *δ* 5.53–5.42 (2H, m), 5.22–5.10 (1H, m), 4.72–4.69 (2H, m), 2.26–2.07 (6H, m), 1.49–1.33 (20H, m), 0.95 (3H, t, *J* = 6.7 Hz); ^13^C NMR (CDCl_3_, 100.62 MHz) *δ* 208.59 (C-2), 132.86 (C-7), 129.21 (C-6), 89.59 (C-3), 74.71 (C-1), 32.02 (C-17), 29.78 (C-5, C-11), 29.75 (C-13, C-9), 29.71 (C-14, C15), 29.60 (C-12), 29.46 (C-16), 29.33 (C-10), 27.78 (C-8), 27.33 (C-4), 22.76 (C-18), 14.10 (C-19); anal. calcd for C_19_H_34_: C, 86.94; H, 13.05; found: C, 86.87; H, 12.97; HRMS (ESI-TOF): calcd for C_19_H_34_ [M + H]^+^263.4812, found 263.4807.

#### 3.11.6. Cross-Cyclomagnesiation of (Z)-alk-1,2,6-trienes (**9a**–**e**) and Allene Alcohol Tetrahydropyran Ethers (**10a**–**d**) with EtMgBr in the Presence of Mg Metal and Cp_2_TiCl_2_ Catalyst (General Procedure)

Diethyl ether (30 mL), (Z)-alk-1,2,6-trienes (**9a**–**e**) (4.8 mmol), allene alcohol tetrahydropyran ethers (**10a**–**d**) (4.0 mmol), EtMgBr (40.0 mmol) (as 1.5 M solution in Et_2_O), Mg powder (0.7 g, 30.0 mmol) and Cp_2_TiCl_2_ (0.1 g, 0.4 mmol) were placed in a glass reactor with stirring under argon (~0 °C). The reaction mixture was warmed to room temperature (20−22 °C) and stirred for 6 h. The reaction mixture was treated with a 5% solution of NH_4_Cl in H_2_O (20 mL) and extracted with Et_2_O (2 × 100 mL). The combined organic phases were dried over MgSO_4_, filtered and the solvents were removed under reduced pressure. Silica gel column chromatography (*n*-hexane/EtOAc (35/1)) of the residue gave compound **12a**–**g** as a pale yellow oily liquid.

##### 2-((5*Z*,9*Z*,13*Z*)-Octadeca-5,9,13-trien-1-yloxy)tetrahydro-2H-pyran (**12a**)

75% yield. ^1^H NMR (CDCl_3_, 400 MHz) *δ* 5.43–5.37 (6H, m), 4.58 (1H, t, *J* = 3.2 Hz), 3.87–3.37 (4H, m), 2.09–2.02 (12H, m), 1.63–1.26 (14H, m), 0.90 (3H, t, *J* = 7.2 Hz,); ^13^C NMR (CDCl_3_, 100.62 MHz) *δ* 130.29 (C-9), 129.95 (C-10), 129.61 (C-5), 129.52 (C-14), 129.39 (C-6), 129.06 (C-13), 98.74 (C-19), 67.42 (C-1), 62.17 (C-23), 31.91 (C-16), 30.74 (C-2), 29.37 (C-20), 27.45 (C-4), 27.40 (C-12), 27.36 (C-7), 27.04 (C-15), 26.94 (C-8, C-11), 26.38 (C-3), 25.51 (C-22), 22.32 (C-17), 19.60 (C-21), 13.96 (C-18); anal. calcd for C_23_H_40_O_2_: C, 78.57; H, 12.32; found: C, 78.50; H, 12.26; HRMS (ESI-TOF): calcd for C_23_H_40_O_2_ [M + H]^+^352.5942, found 352.5936.

##### 2-((5*Z*,9*Z*,13*Z*)-Icosa-5,9,13-trien-1-yloxy)tetrahydro-2H-pyran (**12b**)

80% yield. ^1^H NMR (CDCl_3_, 400 MHz) *δ* 5.41–5.32 (6H, m), 4.60 (1H, t, *J* = 3.2 Hz), 3.91–3.38 (4H, m), 2.11–1.97 (12H, m), 1.65–1.28 (18H, m), 0.90 (3H, t, *J* = 7.2 Hz). ^13^C NMR (CDCl_3_, 100.62 MHz) *δ* 130.43 (C-14), 130.00 (C-5), 129.67 (C-10), 129.57 (C-9), 129.44 (C-6), 129.09 (C-13), 98.82 (C-21), 67.49 (C-1), 62.29 (C-25), 31.79 (C-18), 30.78 (C-2), 29.72 (C-22), 29.40 (C-16), 29.00 (C-17), 27.47 (C-4), 27.42 (C-15), 27.38 (C-12), 27.28 (C-7), 27.08 (C-8, C-11), 26.39 (C-3), 25.52 (C-24), 22.66 (C-19), 19.66 (C-23), 14.11 (C-20); anal. calcd for C_25_H_44_O_2_: C, 79.72; H, 11.77; found: C, 79.67; H, 11.69; HRMS (ESI-TOF): calcd for C_25_H_44_O_2_ [M + H]^+^377.6236, found 377.6230.

##### 2-((5*Z*,9*Z*,13*Z*)-Docosa-5,9,13-trien-1-yloxy)tetrahydro-2H-pyran (**12c**)

78% yield. ^1^H NMR (CDCl_3_, 400 MHz) *δ* 5.43–5.36 (6H, m), 4.59 (1H, t, *J* = 3.2 Hz), 3.89–3.36 (4H, m), 2.13–1.96 (12H, m), 1.64–1.27 (22H, m), 0.89 (3H, t, *J* = 7.2 Hz); ^13^C NMR (CDCl_3_, 100.62 MHz) *δ* 130.36 (C-14), 129.95 (C-5), 129.62 (C-10), 129.52 (C-9),129.40 (C-6), 129.04 (C-13), 98.74 (C-23), 67.43 (C-1), 62.17 (C-27), 31.89 (C-10), 30.75 (C-20), 29.73 (C-2), 29.51 (C-24), 29.38 (C-16), 29.31 (C-17, C-18), 29.23 (C-19), 27.45 (C-4), 27.40 (C-15), 27.36 (C-12), 27.25 (C-7), 27.05 (C-8, C-11), 26.38 (C-3), 25.52 (C-26), 22.66 (C-21), 19.61 (C-25), 14.06 (C-22); anal. calcd for C_27_H_48_O_2_: C, 80.13; H, 11.95; found: C, 80.09; H, 11.87; HRMS (ESI-TOF): calcd for C_27_H_48_O_2_ [M + H]^+^405.6767, found 405.6760.7293.

##### 2-((9*Z*,13*Z*,17*Z*)-Triaconta-9,13,17-trien-1-yloxy)tetrahydro-2H-pyran (**12d**)

83% yield. ^1^H NMR (CDCl_3_, 400 MHz) *δ* 5.42–5.36 (6H, m), 4.59 (1H, t, *J* = 3.2 Hz), 3.91–3.33 (4H, m), 2.12–1.98 (12H, m), 1.63–1.28 (38H, m), 0.89 (3H, t, *J* = 7.2 Hz); ^13^C NMR (CDCl_3_, 100.62 MHz) *δ* 130.39 (C-9), 130.33 (C-18), 129.60 (C-14, C-13), 129.10 (C-10), 129.07 (C-17), 98.80 (C-31), 67.65 (C-1), 62.26 (C-35), 31.95 (C-28), 30.79 (C-32), 29.77 (C-2), 29.71 (C-4, C-22), 29.68 (C-7, C-20, C-24), 29.59 (C-25, C-26), 29.52 (C-6), 29.49 (C-23), 29.38 (C-27), 29.34 (C-21), 29.28 (C-5), 27.46 (C-8, C-19), 27.39 (C-11, C-16), 27.27 (C-12, C-15), 26.26 (C-3), 25.54 (C-34), 22.70 (C-29), 19.66 (C-33), 14.12 (C-30); anal. calcd for C_35_H_64_O_2_: C, 81.32; H, 12.48; found: C, 81.27; H, 12.41; HRMS (ESI-TOF): calcd for C_35_H_64_O_2_ [M + H]^+^517.8894, found 517.8891.

##### 2-((11*Z*,15*Z*,19*Z*)-Triaconta-11,15,19-trien-1-yloxy)tetrahydro-2H-pyran (**12e**)

81% yield. ^1^H NMR (CDCl_3_, 400 MHz) *δ* 5.43–5.36 (6H, m), 4.59 (1H, t, *J* = 3.2 Hz), 3.91–3.37 (4H, m,), 2.10–1.98 (12H, m), 1.64–1.28 (38H, m), 0.90 (3H, t, *J* = 7.2 Hz); ^13^C NMR (CDCl_3_, 100.62 MHz) *δ* 130.39 (C-11, C-20), 129.61 (C-15, C-16), 129.08 (C-12, C-19), 98.81 (C-31), 67.68 (C-1), 62.27 (C-35), 31.94 (C-28), 30.80 (C-2), 29.78 (C-32), 29.77 (C-4), 29.72 (C-7, C-24), 29.68 (C-9, C-22), 29.66 (C-25), 29.62 (C-5), 29.59 (C-6), 29.57 (C-26), 29.52 (C-27), 29.37 (C-8), 29.34 (C-23), 27.47 (C-10, C-21), 27.39 (C-13, C-18), 27.28 (C-14, C-17), 26.27 (C-3), 25.54 (C-34), 22.70 (C-29), 19.69 (C-33), 14.12 (C-30); anal. calcd for C_35_H_64_O_2_: C, 81.32; H, 12.48; found: C, 81.29; H, 12.40; HRMS (ESI-TOF): calcd for C_35_H_64_O_2_ [M + H]^+^517.8894, found 517.8890.

##### 2-((11*Z*,15*Z*,19*Z*)-Dotriaconta-11,15,19-trien-1-yloxy)tetrahydro-2H-pyran (**12f**)

77% yield. ^1^H NMR (CDCl_3_, 400 MHz) *δ* 5.42–5.37 (6H, m), 4.59 (1H, t, *J* = 3.2 Hz), 3.91–3.36 (4H, m), 2.10–1.98 (12H, m), 1.64–1.28 (42H, m), 0.90 (3H, t, *J* = 7.2 Hz); ^13^C NMR (CDCl_3_, 100.62 MHz) *δ* 130.37 (C-11, C-20), 129.59 (C-15, C-16), 129.07 (C-12, C-19), 98.78 (C-33), 67.66 (C-1), 62.23 (C-37), 31.95 (C-30), 30.79 (C-2), 29.77 (C-34), 29.72 (C-4), 29.69 (C-7, C-24), 29.66 (C-9, C-22, C-26), 29.62 (C-27, C-5), 29.58 (C-28, C-6), 29.52 (C-25), 29.39 (C-29), 29.35 (C-8, C-23), 27.47 (C-10, C-21), 27.39 (C-13, C-18), 27.28 (C-14, C-17), 26.28 (C-3), 25.55 (C-36), 22.71 (C-31), 19.68 (C-35), 14.12 (C-32); anal. calcd for C_37_H_68_O_2_: C, 81.55; H, 12.57; found: C, 81.49; H, 12.50; HRMS (ESI-TOF): calcd for C_37_H_68_O_2_ [M + H]^+^545.9425, found 545.9420.

##### 2-((13*Z*,17*Z*,21*Z*)-Dotriaconta-13,17,21-trien-1-yloxy)tetrahydro-2H-pyran (**12g**)

79% yield. ^1^H NMR (CDCl_3_, 400 MHz) *δ* 5.42–5.37 (6H, m), 4.59 (1H, t, *J* = 3.2 Hz), 3.91–3.36 (4H, m), 2.10–1.97 (12H, m), 1.62–1.28 (42H, m), 0.90 (3H, t, *J* = 7.2 Hz). ^13^C NMR (CDCl_3_, 100.62 MHz) *δ* 130.35 (C-13, C-22), 129.58 (C-17, C-18), 129.07 (C-14, C-21), 98.75 (C-33), 67.64 (C-1), 62.18 (C-37), 31.94 (C-30), 30.79 (C-2, C-34), 29.78 (C-4), 29.68 (C-9, C-26), 29.64 (C-5, C-6), 29.60 (C-7, C-11, C-24), 29.53 (C-8, C-27), 29.38 (C-28, C29), 29.35 (C-10, C-25), 27.47 (C-12, C-23), 27.39 (C-15, C-20), 27.28 (C-16, C-19), 26.28 (C-3), 25.55 (C-36), 22.71 (C-31), 19.66 (C-35), 14.11 (C-32); anal. calcd for C_37_H_68_O_2_: C, 81.55; H, 12.57; found: C, 81.48; H, 12.48; HRMS (ESI-TOF): calcd for C_37_H_68_O_2_ [M + H]^+^545.9425, found 545.9419.

#### 3.11.7. General Procedures for the Preparation of 1Z,5Z,9Z-Dienoic Acids **13a**–**g**

To a solution of tetrahydropyran ester (0.5 mmol) in acetone (12 mL) and CH_2_Cl_2_ (2 mL) at room temperature (20–22 °C) Jones reagent (0.5 mL) was added dropwise. The reaction mixture was stirred at room temperature (20–22 °C) for 1 h, then treated with water (2.5 mL), excess solvent was evaporated under vacuum (acetone, CH_2_Cl_2_), the aqueous layer was extracted with diethyl ether, dried over MgSO_4_. The reaction product was isolated by column chromatography (silica gel, Acrus (0.060–0.200 mm), eluent petroleum ether/ethyl acetate) [43,44].

##### (5*Z*,9*Z*,13*Z*)-Octadeca-5,9,13-trienoic acid (**13a**)

77% yield. IR (KBr) ν_max_ 3524, 2900, 2866, 1740, 1722, 1665, 1463, 1384, 1365, 1250, 1241, 1034, 737 cm^−1^; ^1^H NMR (CDCl_3_, 400 MHz) *δ* 5.43–5.38 (6H, m), 2.42–2.35 (2H, m), 2.13–2.05 (12H, m), 1.76–1.69(2H, m), 1.36–1.28 (4H, m), 0.92 (3H, t, *J* = 6.6 Hz); ^13^C NMR (CDCl_3_, 100.62 MHz) *δ* 179.63 (C-1), 130.56 (C-6), 130.39 (C-9), 129.80 (C-10), 129.40 (C-14), 129.08 (C-5),128.65 (C-13), 33.36 (C-2), 31.93 (C-16), 27.46 (C-7, C-12), 27.36 (C-15), 27.32 (C-4), 26.97 (C-11), 26.49 (C-8), 24.58 (C-3), 22.35 (C-17), 13.99 (C-18); anal. calcd for C_18_H_30_O_2_: C, 77.64; H, 10.86; found: C, 77.58; H, 10.80; HRMS (ESI-TOF): calcd for C_18_H_30_O_2_ [M + H]^+^279.4375, found 279.4370.

##### (5*Z*,9*Z*,13*Z*)-Icosa-5,9,13-trienoic acid (**13b**)

79% yield. IR (KBr) ν_max_ 3378, 3045, 2930, 2838, 1780, 1740, 1651, 1465, 1367, 1242, 1130, 748 cm^−1^; ^1^H NMR (CDCl_3_, 400 MHz) *δ* 5.95.34 (6H, m), 2.47–2.35 (2H, m), 2.15–1.97 (12H, m), 1.76–1.62 (8H, m), 1.42–1.28 (2H, m),0.91 (3H, t, *J* = 6.6 Hz); ^13^C NMR (CDCl_3_, 100.62 MHz) *δ* 179.88 (C-1), 130.57 (C-6), 130.45 (C-9), 129.81 (C-10), 129.40 (C-14), 129.07 (C-5), 128.65 (C-13), 33.40 (C-2), 31.79 (C-18), 29.71 (C-16), 29.00 (C-17), 27.46 (C-7), 27.36 (C-12), 27.32 (C-15), 27.28 (C-4), 26.49 (C-8, C-11), 24.58 (C-3), 22.66 (C-19), 14.10 (C-20); anal. calcd for C_20_H_34_O_2_: C, 78.37; H, 11.18; found: C, 78.30; H, 11.11; HRMS (ESI-TOF): calcd for C_20_H_34_O_2_ [M + H]^+^307.4907, found 307.4900.

##### (5*Z*,9*Z*,13*Z*)-Docosa-5,9,13-trienoic acid (**13c**)

81% yield. IR (KBr) ν_max_ 3520, 2918, 2865, 1748, 1735, 1670, 1463, 1390, 1358, 1253, 1168, 1030, 752 cm^−1^; ^1^H NMR (CDCl_3_, 400 MHz) *δ* 5.46–5.35 (6H, m), 2.42–2.35 (2H, m), 2.15–2.02 (12H, m), 1.76–1.62 (12H, m), 1.40–1.28 (2H, m), 0.90 (3H, t, *J* = 6.6 Hz); ^13^C NMR (CDCl_3_, 100.62 MHz) *δ* 179.82 (C-1), 130.56 (C-6), 130.45 (C-9), 129.81 (C-10), 129.39 (C-14), 129.07 (C-5), 128.65 (C-13), 33.39 (C-2), 31.91 (C-20), 29.75 (C-16), 29.53 (C-17, C-18), 29.33 (C-19), 29.06 (C-16), 27.46 (C-7), 27.36 (C-12), 27.32 (C-15), 27.28 (C-4), 26.49 (C-8, C-11), 24.58 (C-3), 22.69 (C-21), 14.11 (C-22); anal. calcd for C_22_H_38_O_2_: C, 78.98; H, 11.44; found: C, 78.91; H, 11.37; HRMS (ESI-TOF): calcd for C_22_H_38_O_2_ [M + H]^+^335.5438, found 335.5433.

##### (9*Z*,13*Z*,17*Z*)-Triaconta-9,13,17-trienoic acid (**13d**)

76% yield. IR (KBr) ν_max_ 3520, 2920, 2865, 1730, 1670, 1463, 1378, 1330, 1248, 1158, 1025, 736 cm^−1^; ^1^H NMR (CDCl_3_, 400 MHz) *δ* 5.42–5.36 (6H, m), 2.37 (2H, t), 2.12–2.02 (12H, m), 1.67–1.62 (28H, m), 1.42–1.28 (2H, m), 0.91 (3H, t, *J* = 6.6 Hz); ^13^C NMR (CDCl_3_, 100.62 MHz) *δ* 179.95 (C-1), 130.43 (C-10), 130.25 (C-14), 129.65 (C-13), 129.60 (C-18), 129.22 (C-9), 129.09 (C-17), 34.02 (C-2), 31.95 (C-28), 29.77 (C-22), 29.71 (C-7), 29.68 (C-20, C-24, C-25), 29.59 (C-23, C-26), 29.38 (C-27), 29.35 (C-21), 29.17 (C-5), 29.09 (C-4), 29.05 (C-6), 27.48 (C-8, C-19), 27.45 (C-11), 27.39 (C-16), 27.29 (C-12), 27.23 (C-15), 24.67 (C-3), 22.71 (C-29), 14.13 (C-30); anal. calcd for C_30_H_54_O_2_: C, 80.65; H, 12.18; found: C, 80.60; H, 12.11; HRMS (ESI-TOF): calcd for C_30_H_54_O_2_ [M + H]^+^447.7565, found 447.7560.

##### (11*Z*,15*Z*,19*Z*)-Triaconta-11,15,19-trienoic acid (**13e**)

81% yield. IR (KBr) ν_max_ 3360, 3035, 1854, 1718, 1640, 1564, 1478, 1360, 1265, 1018, 735 cm^−1^; ^1^H NMR (CDCl_3_, 400 MHz) *δ* 5.43–5.36 (6H, m), 2.36 (2H, t), 2.11–1.98 (12H, m), 1.70–1.59 (28H, m), 1.38–1.28 (2H, m), 0.90 (3H, t, *J* = 6.6 Hz); ^13^C NMR (CDCl_3_, 100.62 MHz) *δ* 180.06 (C-1), 130.42 (C-11), 130.35 (C-20), 129.62 (C-15, C-16), 129.13 (C-12), 129.08 (C-19), 34.07 (C-2), 31.93 (C-28), 29.76 (C-7), 29.74 (C-24), 29.66 (C-9, C-22), 29.58 (C-25), 29.50 (C-6), 29.42 (C-26), 29.37 (C-27), 29.34 (C-8), 29.29 (C-23), 29.25 (C-5), 29.08 (C-4), 27.47 (C-10, C-21), 27.43 (C-13), 27.39 (C-18), 27.27 (C-14, C-17), 24.69 (C-3), 22.70 (C-29), 14.11 (C-30); anal. calcd for C_30_H_54_O_2_: C, 80.65; H, 12.18; found: C, 80.59; H, 12.10; HRMS (ESI-TOF): calcd for C_30_H_54_O_2_ [M + H]^+^447.7565, found 447.7561.

##### (11*Z*,15*Z*,19*Z*)-Dotriaconta-11,15,19-trienoic acid (**13f**)

77% yield. IR (KBr) ν_max_ 3360, 3030, 1850, 1718, 1642, 1470, 1365, 1263, 1020, 742 cm^−1^; ^1^H NMR (CDCl_3_, 400 MHz) *δ* 5.44–5.38 (6H, m), 2.37 (2H, t), 2.14–2.02 (12H, m), 1.69–1.61 (32H, m), 1.39–1.28 (2H, m), 0.91 (3H, t, *J* = 6.6 Hz); ^13^C NMR (CDCl_3_, 100.62 MHz) *δ* 180.22 (C-1), 130.41 (C-11), 130.35 (C-20), 129.62 (C-15, C-16), 129.13 (C-12), 129.08 (C-19), 34.09 (C-2), 31.95 (C-30), 29.77 (C-7), 29.72 (C-24), 29.68 (C-9, C22), 29.60 (C-26, C-27), 29.50 (C-28), 29.43 (C-6), 29.38 (C-25), 29.35 (C-29), 29.30 (C-8), 29.26 (C-23), 29.08 (C-4), 27.47 (C-10, C-21), 27.40 (C-13, C-18), 27.28 (C-14, C-17), 24.68 (C-3), 22.71 (C-31), 14.12 (C-32); anal. calcd for C_32_H_58_O_2_: C, 80.94; H, 12.31; found: C, 80.87; H, 12.28; HRMS (ESI-TOF): calcd for C_32_H_58_O_2_ [M + H]^+^475.8096, found 475.8091.

##### (13*Z*,17*Z*,21*Z*)-Dotriaconta-13,17,21-trienoic acid (**13g**)

82% yield. IR (KBr) ν_max_ 3360, 3030, 1852, 1720, 1646, 1474, 1360, 1265, 1020, 741 cm^−1^; ^1^H NMR (CDCl_3_, 400 MHz) *δ* 5.47–5.38 (6H, m), 2.36 (2H, t), 2.12–2.02 (12H, m), 1.70–1.62 (32H, m), 1.46–1.30 (2H, m), 0.91 (3H, t, *J* = 6.6 Hz); ^13^C NMR (CDCl_3_, 100.62 MHz) *δ* 180.19 (C-1), 130.39 (C-13, C-22), 129.62 (C-17, C-18), 129.09 (C-14, C-21), 34.11 (C-2), 31.94 (C-30), 29.77 (C-9, C-26), 29.68 (C-11, C-24), 29.62 (C-8, C-27), 29.61 (C-6), 29.46 (C-7), 29.38 (C-28, C-29), 29.35 (C-10, C-25), 29.27 (C-5), 29.09 (C-4), 27.47 (C-12, C-23), 27.40 (C-15), 27.39 (C-20), 27.29 (C-16, C-19), 24.70 (C-3), 22.71 (C-31), 14.11 (C-32); anal. calcd for C_32_H_58_O_2_: C, 80.94; H, 12.31; found: C, 80.89; H, 12.25; HRMS (ESI-TOF): calcd for C_32_H_58_O_2_ [M + H]^+^475.8096, found 475.8090.

## 4. Conclusions

Compound **13c** exhibits a pronounced antitumor effect due to the multi-target effect on the main intracellular targets. The levels of all main kinases responsible for cell growth and proliferation markedly decrease under the action of the synthesized trienoic acid **13c**; simultaneously, acid **13c** actively inhibits topoisomerase I and induces apoptosis of tumor cells according to the intrinsic mitochondrial pathway. Using molecular docking, we found that the preferable binding sites for the carboxylate group in the acids **13a**–**g** are the K443, K587 and N722 residues. Among the activated signaling pathways, the phosphorylation levels of ERK1/2, Akt, JNK and p38 were characterized by different basic kinetics in response to the action of the test compound, but differed in lower values than in the control sample. Interestingly, STAT-3 and STAT-5 phosphorylation, characterized by a progressive increase over time in untreated cultures, was inhibited in samples containing the test compound. In general, these results are consistent with the results of our earlier studies of higher fatty acids with diene and triene groups, showing the ability to suppress these signaling pathways in various tumor cultures. The synthesized trienoic acids can claim the role of multitarget compounds that are able to simultaneously penetrate through various biological membranes, including mitochondrial, uncouple oxidation and phosphorylation, thereby reducing mitochondrial potential, as well as inhibiting topoisomerase I and affecting the main signaling pathways of cell proliferation.

## Figures and Tables

**Table 1:** Cytotoxic activity in vitro of trienoic acids **13a**–**g** (48 h).

Acids	Jurkat, IC_50_, µM	K562, IC_50_, µM	U937, IC_50_, µM	HL60, IC_50_, µM	HEK293, IC_50_, µM	HeLa, IC_50_, µM	Fibroblasts, IC_50_, µM
**13a**	0.178 ± 0.022	0.211 ± 0.021	0.165 ± 0.019	0.159 ± 0.014	0.617 ± 0.054	0.574 ± 0.052	1.119 ± 0.104
**13b**	0.147 ± 0.015	0.172 ± 0.016	0.139 ± 0.013	0.127 ± 0.011	0.523 ± 0.049	0.489 ± 0.041	0.937 ± 0.086
**13c**	0.105 ± 0.009	0.136 ± 0.012	0.098 ± 0.010	0.086 ± 0.008	0.451 ± 0.039	0.311 ± 0.027	0.754 ± 0.069
**13d**	0.448 ± 0.043	0.479 ± 0.046	0.456 ± 0.041	0.409 ± 0.037	1.297 ± 0.114	1.029 ± 0.091	1.738 ± 0.154
**13e**	0.421 ± 0.038	0.467 ± 0.043	0.394 ± 0.037	0.387 ± 0.039	1.082 ± 0.093	0.983 ± 0.086	1.597 ± 0.139
**13f**	0.454 ± 0.046	0.471 ± 0.051	0.461 ± 0.043	0.404 ± 0.040	1.211 ± 0.106	1.087 ± 0.094	1.914 ± 0.176
**13g**	0.122 ± 0.011	0.154 ± 0.014	0.117 ± 0.010	0.104 ± 0.009	0.487 ± 0.046	0.354 ± 0.032	0.739 ± 0.068

Results are expressed as mean ± SE (*n* = 3). Data are presented as mean plus standard error *p* > 0.01.

**Table 2:** Inhibitory activity of trienoic acids **13a**–**g** in vitro.

Acids	13a	13b	13c	13d	13e	13f	13g
hTopI inhibition, µM	0.2	0.2	0.1	0.8	0.6	0.8	0.4

## Data Availability

The data presented in this study are available in this article.
